# Global trends in preclinical and clinical undergraduate endodontic education: A worldwide survey

**DOI:** 10.1038/s41598-025-94836-y

**Published:** 2025-03-24

**Authors:** Raidan Ba-Hattab, Nessrin A. Taha, Muna M. Shaweesh, Paulo J. Palma, Saleem Abdulrab

**Affiliations:** 1 Pre-Clinical Oral Health Sciences Department, College of Dental Medicine, QU Health, Qatar University, Doha, Qatar; 2 Department of Conservative Dentistry, Faculty of Dentistry, Jordan University of Science and Technology, Irbid, Jordan; 3 Al Thumamah Health Center, Primary Health Care Corporation, Doha, Qatar; 4 Faculty of Medicine, Center for Innovation and Research in Oral Sciences (CIROS), University of Coimbra, Coimbra, Portugal; 5 Faculty of Medicine, Institute of Endodontics, University of Coimbra, Coimbra, Portugal; 6 Al Khor Health Center, Primary Health Care Corporation, Doha, Qatar

**Keywords:** Endodontics, Endodontic curriculum, Endodontic education, Preclinical, Clinical, Dental education, Dental treatments

## Abstract

This is a global study that evaluated the undergraduate endodontic curriculum in dental schools worldwide. An online questionnaire sent to faculty members involved in undergraduate endodontic teaching at 50 dental institutions across six continents. The questionnaire was a modified version of previously validated survey comprised of 46 questions in three sections on essential aspects of the didactic, preclinical (PT), clinical trainings (CT), and assessments in undergraduate endodontic programs. The response rate was 76%. 63.2% of schools have a separate preclinical course, with 65.8% having a dedicated clinical area. Traditional methods dominate, with 84.2% using phantom heads and 78.9% using natural extracted teeth. The student-to-staff ratio for clinical training ranges from 1:6 to 1:9, with most supervisors specializing in endodontics (76.3%). Lectures and practical sessions are the primary teaching methods. Non-surgical root canal treatment (RCT) is the most common procedure (94.7%), and the majority use stainless steel or rotary nickel-titanium files. Cold lateral compaction is the preferred obturation technique globally (81.6%), except in North America. Advanced technologies like magnifying systems and ultrasonic instruments are rarely used in training. The conclusion emphasizes the need for global standardization and adoption of modern techniques to enhance the quality of endodontic education and care.

**Supplementary Information:**

The online version contains supplementary material available at 10.1038/s41598-025-94836-y.

## Introduction

Irreversible pulpitis and apical periodontitis are frequent dental emergencies among adults^1^. Such dental emergencies are managed mainly by general dental practitioners (GDPs), who either relieve pain, or perform root canal treatment (RCT) aiming to preserve the tooth^2^. High-quality care depends on accurate diagnosis and decision-making, based on a good foundation of knowledge of the aetiology of pulpal and periapical diseases. That’s why GDPs should know well when to treat, why to treat, what to treat, and how to treat^3^.

Previous reports in the United Kingdom (UK) showed that the quality of RCT performed by GDPs and undergraduate students was consistently below the acceptable levels defined by the European Society of Endodontology (ESE)^4–7^. The insufficient acquisition of knowledge and technical skills during undergraduate training is one possible reason for these findings. Therefore, the ESE and the Association for Dental Education in Europe (ADEE) in collaboration with the American Association of Endodontists have established guidelines to enhance undergraduate endodontic curricula, recommending the inclusion of contemporary technologies and materials^8–10^.

The ESE guidelines for the undergraduate curriculum formed a benchmarking reference for dental schools and regulatory bodies. Initially, these guidelines were focused on the curriculum’s content, defining recommended levels of competence. The latest edition (2024) adapted a list of expected achieved capabilities of the graduating students; to provide a minimum level of competency, emphasizing quality over quantity in CT^3^. By the end of their training, students should possess self-efficacy, which is the belief and self-assurance that practitioner has the capability to perform specific tasks successfully, despite any existing realities^11,12^.

The poor technical quality of RCTs performed by GDPs reported in the above studies highlighted the need for assessment of undergraduate endodontic curriculum, which may be the root cause of this issue. An evaluation of endodontic education across 16 UK dental schools found that training has improved and become more consistent over the last 20 years^13^. In Spain, dental schools following ESE guidelines reported better outcomes in private institutions than public ones^8,9^. However, recommendations include increasing the use of magnification and ultrasonic instruments^14^. A study in Saudi Arabia similarly found consistent endodontic curriculum content and delivery across various dental schools^15^.

So far, there is no study that evaluated undergraduate endodontic education globally. Therefore, the objective of this study was to explore the status of undergraduate endodontic teaching among dental schools around the world.

## Results

**Fig. 1:**
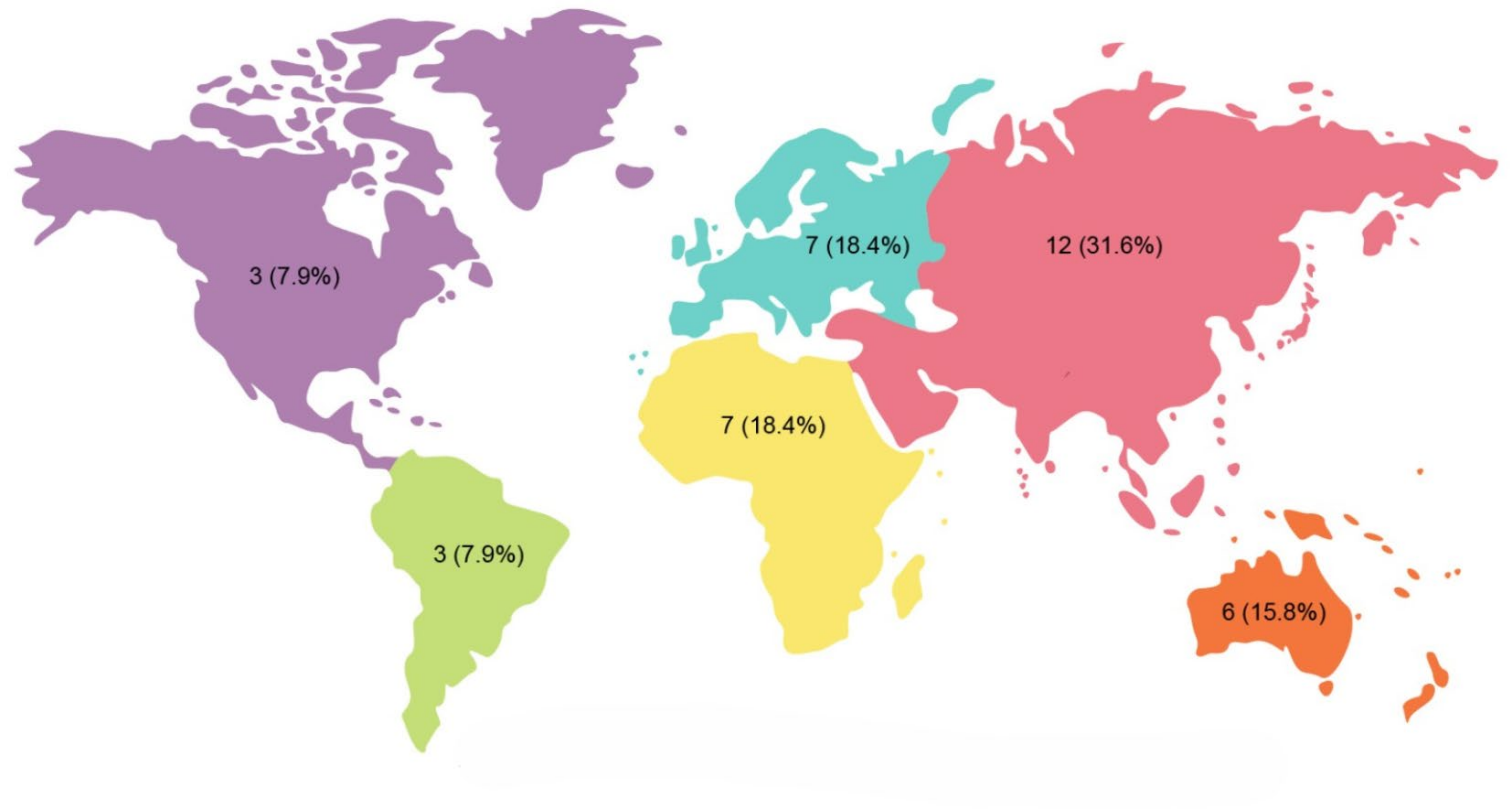
Distribution of respondents across the continents.

This study provides the first global overview of undergraduate endodontic teaching, encompassing data from thirty-eight dental schools across six continents. The response rate was 76%. Figure 1 shows the distribution of the respondents based on their geographical location.

The preclinical endodontic course is taught as an integral part of other courses in Asia, Africa, and North America; however, it is taught as a separate course in South America and Australia. The majority of dental schools have specific areas in the clinic designated for endodontic training; nevertheless, half of the European schools offer CT in comprehensive dental clinics (Table 1).

**Table 1:** Delivery of the preclinical courses (separate or integrated), allocation of clinical area for CT, types of dental simulator and root canals used in the PT (n, %).

	Asia (*n* = 12)	Africa (*n* = 7)	Europe (*n* = 7)	Australia (*n* = 6)	North America (*n* = 3)	South America (*n* = 3)	Total (*n* = 38)
Is preclinical course taught as a separate course or in integration with other courses?							
It is a separate course	10 (83.3%)	4 (57.1%)	5 (71.4%)	2 (33.3%)	3 (100.0%)	0 (0.0%)	24 (63.2%)
It is integrated with other courses	2 (16.7%)	3 (42.9%)	2 (28.6%)	4 (66.7%)	0 (0.0%)	3 (100.0%)	14 (36.8%)
**Is there a clinical area specifically assigned to Endodontic?**							
Yes	8 (66.7%)	4 (57.1%)	3 (42.9%)	5 (83.3%)	3 (100.0%)	2 (66.7%)	25 (65.8%)
No	4 (33.3%)	3 (42.9%)	4 (57.1%)	1 (16.7%)	0 (0.0%)	1 (33.3%)	13 (34.2%)
**Types of dental simulators available for the students in the PT training**							
Traditional phantom head	11 (91.7%)	5 (71.4%)	7 (100.0%)	5 (83.3%)	3 (100.0%)	1 (33.3%)	32 (84.2%)
Computer-supported and Virtual reality dental simulators	2 (16.7%)	1 (14.3%)	1 (14.3%)	0 (0.0%)	0 (0.0%)	2 (66.7%)	6 (15.8%)
No dental simulator	0 (0.0%)	1 (14.3%)	1 (14.3%)	1 (16.7%)	0 (0.0%)	0 (0.0%)	3 (7.9%)
Others (Extracted teeth)	0 (0.0%)	0 (0.0%)	0 (0.0%)	0 (0.0%)	0 (0.0%)	1 (33.3%)	1 (2.6%)
**Types of root canals used in PT**							
Canals in natural extracted teeth	12 (100.0%)	7 (100.0%)	4 (57.1%)	2 (33.3%)	3 (100.0%)	2 (66.7%)	30 (78.9%)
Canals in commercial plastic teeth	4 (33.3%)	2 (28.6%)	3 (42.9%)	4 (66.7%)	2 (66.7%)	1 (33.3%)	16 (42.1%)
Canals in 3D printed teeth	2 (16.7%)	1 (14.3%)	2 (28.6%)	3 (50.0%)	0 (0.0%)	1 (33.3%)	9 (23.7%)
Canals in acrylic blocks with simple curves	4 (33.3%)	0 (0.0%)	3 (42.9%)	2 (33.3%)	2 (66.7%)	0 (0.0%)	11 (28.9%)
Canals in acrylic blocks with S-shaped curves	0 (0.0%)	0 (0.0%)	1 (14.3%)	0 (0.0%)	1 (33.3%)	0 (0.0%)	2 (5.3%)

PT: Preclinical training, CT: clinical training.

### Types of dental simulators and teeth available for the PT

All dental schools in Europe and North America (100%), and most dental schools in Australia, Africa, and Asia (> 70%), employ the traditional manikin in the PT. Additionally, one school in South America uses extracted teeth but does not utilize either the traditional phantom head or digital VR. A more advanced method is used by 15% of the schools is the virtual reality simulators; in Asia (16%), Africa (14%), Europe (14%), and South America (66%). Three schools (7.9%) have no dental simulators, one in Europe, Australia, and Africa (Table 1). Note: The type of simulator (traditional or digital) is independent of the canal material (extracted human teeth or commercial plastic teeth).

Table 1 also shows types of root canals used in the PT, regardless of the type of dental simulator with some schools utilizing multiple tooth types. Extracted human teeth were used at the majority of dental schools worldwide (78.9%). However, some schools are using canals in commercial plastic teeth (42.1%); most of these schools are in Australia and North America, with 66.7% for both regions.

### Academic years involving PT and CT

All dental schools in North America and over 50% of the schools in Europe, Australia, and South America offer PT in the second year. Two-thirds of African schools and more than half of Asian schools teach PT in the third and fourth years, respectively. Further, more than half the schools in Europe and Australia offer the course in the third year as well. Regarding CT, most Australian, European, North American, and South American schools offer CT in the third and fourth years, while Asian and African schools offer them in the fourth and fifth years (Supplementary Table S1).

### Total hours allocated to the theoretical part of endodontic training

The total hours allocated for teaching the theoretical part of preclinical endodontics ranged from 15 to 90 h (average 52.5 h). Ninety hours are required in total to teach the endodontics theory in all North American schools, while 50% of Australian and Asian schools allocate 45 and 90 h, respectively. In Africa, Europe, and South Africa, the time is inconsistent across different schools (Supplementary Table S2).

### Students and staff

The number of students enrolled in endodontic courses varied across schools in different regions, ranging from ≤ 14 (7.9%) to more than 104 students (21.1%). 50% of Australian dental schools enroll 90–104 students, which is much more than dental schools in Asia, Africa, Europe, North and South America (Supplementary Table S2).

Table 2 shows the mean and range of staff: student ratios in each geographical area in the PT and CT training. In the PT, the lowest mean staff: student was in Asia, Europe, and Australia (1:9), and the highest was in South America (1:7). In the CT, Asia, Europe, and Australia had the highest mean ratio (1:6).

**Table 2:** Staff: students ratio and staff qualification (n, %).

	Asia (*n* = 12)		Africa (*n* = 7)		Europe (*n* = 7)		Australia (*n* = 6)		North America (*n* = 3)		South America (*n* = 3)			
**Staff: student ratio**														
	**PT**	**CT**	**PT**	**CT**	**PT**	**CT**	**PT**	**CT**	**PT**	**CT**	**PT**	**CT**		
**Mean**	1:9	1:6	1:8	1:7	1:9	1:6	1:9	1:6	1:8	1:8	1:7	1:8		
**Range**	1:5 − 1:10	1:4 − 1:10	1:5 − 1:10	1:5 − 1:10	1:5 − 1:10	1:3 − 1:10	1:8 − 1:10	1:4 − 1:8	1:8	1:5 − 1:10	1:5 − 1:10	1:5 − 1:10		
**Qualification of the majority of course contributors (n**,** %)**														
	**Asia** (*n* = 12)		**Africa** (*n* = 7)		**Europe** (*n* = 7)		**Australia** (*n* = 6)		**North America** (*n* = 3)		**South America** (*n* = 3)		**Total** (*n* = 38)	
	**PT**	**CT**	**PT**	**CT**	**PT**	**CT**	**PT**	**CT**	**PT**	**CT**	**PT**	**CT**	**PT**	**CT**
General dentistry	0 (0.0%)	0 (0.0%)	1 (14.3%)	1 (14.3%)	1 (14.3%)	1 (14.3%)	2 (33.3%)	2 (33.3%)	0 (0.0%)	0 (0.0%)	2 (66.7%)	2 (66.7%)	6 (15.8%)	6 (15.8%)
General dentistry with particular interest in endodontics	1 (8.3%)	1 (8.3%)	2 (28.6%)	4 (57.1%)	3 (42.9%)	1 (14.3%)	5 (83.3%)	5 (83.3%)	2 (66.7%)	0 (0.0%)	0 (0.0%)	0 (0.0%)	13 (34.2%)	11 (28.9%)
Restorative Dentistry	4 (33.3%)	5 (41.7%)	3 (42.9%)	1 (14.3%)	2 (28.6%)	4 (57.1%)	0 (0.0%)	0 (0.0%)	0 (0.0%)	0 (0.0%)	0 (0.0%)	0 (0.0%)	9 (23.7%)	10 (26.3%)
Exclusively Endodontics	10 (83.3%)	11 (91.7%)	4 (57.1%)	4 (57.1%)	4 (57.1%)	6 (85.7%)	2 (33.3%)	4 (66.7%)	1 (33.3%)	3 (100.0%)	1 (33.3%)	1 (33.3%)	22 (57.9%)	29 (76.3%)

PT: Preclinical training, CT: clinical training.

Regarding the qualification of the majority of the staff contributors, most dental schools in Asia have only Endodontists contributing to PT teaching (83.3%), however, 41.6% of dental schools involve (GDPs) with particular interest in endodontics, and restorative dentistry specialists. GDPs and GDPs with particular interest in Endodontics are involved in more than half of the schools in South America (66.7%), and North America (66.7%), respectively. Other dental schools across various countries have Restorative dentistry specialists, GDPs, and/or GDPs with particular interest in Endodontics. In CT, the majority of the staff supervising undergraduate endodontic treatment were Endodontists in the majority of the responding schools, except in South Africa, where more than two-thirds of the schools had GDPs (Table 2).

### Teaching methods and subjects taught

Seminars are more common in Africa, Europe, Australia, and South America compared to Asia and North America. In particular, Australian and African schools commonly use problem-based learning. Most schools globally, except in Africa, provide additional materials for self-directed learning (Supplementary Table S3).

Regarding the subjects taught in undergraduate endodontic program, Supplementary Table S3 shows a consensus among North American schools on endodontic topics. The majority of the schools taught endodontics in the second year of the dental program. Topics like root canal anatomy and pulp histology, physiopathology, microbiology, radiology, materials, vital pulp therapies, RCT of immature teeth, non-surgical endodontic treatment (RCT), root canal retreatments (RRCTs), restoration of endodontically treated teeth, and dental trauma were taught in the second year in the majority of North American and European schools, and in the third year in Australian schools. Many endodontics topics are introduced to the students in their first academic year in South American universities. In contrast, most of these topics are taught in the fourth and fifth years in African schools and in fifth year in Asian schools.

### Endodontic procedures and minimal requirements

Table 3 shows that there is a consensus among the majority of dental schools regarding performing non-surgical RCT and vital pulp therapy (94.7% and 76.3%), respectively. All respondent schools in North America and more than two-thirds in Australia performed bleaching of endodontically treated teeth. Retreatment is required in half of African schools.

**Table 3:** Types of endodontic treatments the students performing during CT (n, %).

	Asia (*n* = 12)	Africa (*n* = 7)	Europe (*n* = 7)	Australia (*n* = 6)	North America (*n* = 3)	South America (*n* = 3)	Total (*n* = 38)
Non-surgical root canal treatment	11 (91.7%)	6 (85.7%)	7 (100.0%)	6 (100.0%)	3 (100.0%)	3 (100.0%)	36 (94.7%)
Vital pulp therapy	9 (75.0%)	6 (85.7%)	5 (71.4%)	4 (66.7%)	3 (100.0%)	2 (66.7%)	29 (76.3%)
Surgical root canal treatment	0 (0.0%)	0 (0.0%)	1 (14.3%)	0 (0.0%)	0 (0.0%)	0 (0.0%)	1 (2.6%)
Regenerative endodontics	2 (16.7%)	0 (0.0%)	0 (0.0%)	0 (0.0%)	0 (0.0%)	1 (33.3%)	3 (7.9%)
Bleaching of endodontically treated teeth	5 (41.7%)	1 (14.3%)	2 (28.6%)	4 (66.7%)	3 (100.0%)	1 (33.3%)	16 (42.1%)
Non-surgical root canal retreatments	5 (41.7%)	4 (57.1%)	3 (42.9%)	2 (33.3%)	0 (0.0%)	1 (33.3%)	15 (39.5%)
Students do not execute any treatment	0 (0.0%)	0 (0.0%)	0 (0.0%)	0 (0.0%)	0 (0.0%)	0 (0.0%)	0 (0.0%)

CT: clinical training.

Concerning the minimum number of canals for primary RCT (Supplementary Table S4), more than half of dental schools in all geographical areas required the students to complete a minimum of two RCTs for each tooth type in the simulation lab. In the CT, the majority of the schools required the same before graduation, except Australian schools, which did not set a minimum number of RCTs. Further, more than half of dental schools did not require RRCT.

### Endodontic instruments, materials, and techniques (Supplementary tables S5 and S6)

Over 50% of Asian and African schools did not employ magnification systems during the PT or CT schools. Nonetheless, dental loupes were utilized by students in more than half of the responding Australian and European schools. Microscopes and dental loupes were used in two-thirds of North American. Among the three respondents from South American universities, one does not use a magnification system, one uses dental loupes, and another utilizes a microscope. The majority of the schools are not using ultrasonic in PT and CT (94.7% and 76.3%, respectively).

In the PT, over 50% of dental schools worldwide, excluding Europe, exclusively employed electronic apex locators to determine working lengths. In Europe, 42.9% of dental schools utilize radiographs and electronic apex locators. In CT, most schools across various regions using both radiographs and electronic apex locators.

In both PT and CT, manual stainless-steel instruments are predominately used in all geographical areas except North America (33.3%), where all schools use engine driven NiTi (100%). In CT, more than half of dental schools use engine NiTi instruments, except for South American dental schools, which rely solely on manual instruments (100%).

Most schools are incorporating more than two techniques for root canal preparation in PT and CT. The step-back technique is used in more than half of the respondents in Asia, Africa, Europe, and South America. 66.7% of dental schools in South America are also incorporating another manual technique, the standardized technique. African schools are in favor of the crown-down technique (57%).

RRCT is not required in most dental schools in the PT and CT (81.6% and 57.9%, respectively). When RRCT is conducted in PT (in 18.4% of all respondents, the most commonly used instruments in preclinical training include Gates Glidden drills (15.8%), Hedstrom files (10.5%), and nickel-titanium rotary files (10.5%), with limited use of chemical solvents like Xylene, eucalyptol and chloroform (2.6–7.9%). When RRCT is performed in CT (42.1%), students rely on Gates Glidden drills (26.3%), nickel-titanium rotary files (34.2%), and Hedstrom files (26.3%). Additionally, chemical solvents like chloroform, xylene, and eucalyptol are sometimes used (7.9–18.4%).

Almost all institution uses the passive needle irrigation technique in the PT (89.5%) and CT (84.2%). The most commonly used irrigation in PT in all schools (68.4%) is sodium hypochlorite (NaOCl), except in Australia, where they irrigate with water (83%). 58.3% of Asian schools and 57% of African schools are advocating the use of additional solutions such as Normal Saline and Ethylenediaminetetraacetic acid (EDTA), respectively. In the CT, NaOCl is almost used by all schools worldwide (97.4%), followed by EDTA (65.8%). Additionally, more than half of European and south American schools are advocating the use of Chlorhexidine(CHX).

In the PT and CT, the cold lateral condensation technique remained the principle undergraduate obturation technique in all regions, except North America, where they adopt various cold and warm obturation techniques. Half of Asian, Australian, and South American schools advocated the single cone obturation method.

In PT, most respondents in Africa, Europe, and North America did not use Advanced materials and/or technologies (Table 4). In CT, over half of dental schools in Asia, Africa, and South America used Mineral Trioxide Aggregate (MTA). In Australian, over half of the respondents, used Cone beam computed tomography (CBCT) and thermally treated nickel-titanium files.

**Table 4:** Difficulty assessment form and degree of case complexity (n, %).

	Asia (*n* = 12)	Africa (*n* = 7)	Europe (*n* = 7)	Australia (*n* = 6)	North America (*n* = 3)	South America (*n* = 3)	Total (*n* = 38)
Difficulty assessment form							
Yes, there is a difficulty assessment form (the American Association of Endodontics (AAE) difficulty assessment form	1 (8.3%)	3 (42.9%)	2 (28.6%)	3 (50.0%)	2 (66.7%)	0 (0.0%)	11 (28.9%)
Yes, there is a difficulty assessment form (not AAE form).	3 (25.0%)	1 (14.3%)	0 (0.0%)	2 (33.3%)	1 (33.3%)	0 (0.0%)	7 (18.4%)
No special difficulty assessment form.	8 (66.7%)	3 (42.9%)	5 (71.4%)	1 (16.7%)	0 (0.0%)	3 (100.0%)	20 (52.6%)
No	0 (0.0%)	0 (0.0%)	0 (0.0%)	0 (0.0%)	0 (0.0%)	0 (0.0%)	0 (0.0%)
Others	0 (0.0%)	0 (0.0%)	0 (0.0%)	0 (0.0%)	0 (0.0%)	0 (0.0%)	0 (0.0%)
**Degree of case complexity (according to the AAE) that allowed for the students to perform endodontic treatment in the clinic**							
Uncomplicated cases with low difficulty	12 (100.0%)	5 (71.4%)	4 (57.1%)	5 (83.3%)	3 (100.0%)	3 (100.0%)	32 (84.2%)
Cases with moderate difficulty	4 (33.3%)	5 (71.4%)	5 (71.4%)	4 (66.7%)	1 (33.3%)	2 (66.7%)	21 (55.3%)
Cases with high difficulty	1 (8.3%)	0 (0.0%)	0 (0.0%)	1 (16.7%)	0 (0.0%)	1 (33.3%)	3 (7.9%)

Regarding the use of intracanal medicaments, all African schools advocated the use of intracanal dressing in the PT, while most of the respondents did not. In the CT, most of the respondents are placing Calcium hydroxide (Ca (OH)_2_) routinely between appointments.

While most European, South, and North American respondents are placing a temporary restoration after completing RCT in CT, Asian, African, and Australian respondents are using adhesive restoration after endodontic treatment.

### Difficulty assessment form and degree of case complexity

While more than half of the Australian and North American respondents are following the AAE difficulty assessment form before assigning clinical cases to students, schools in Asia, Africa, and South America didn’t use any specialty form to assess the difficulty of the cases. Among European respondents, only 2 out of 7 universities (28.6%) follow the AAE Difficulty Assessment Form, while the remaining 5(71.4%) do not use any specific difficulty assessment form (Table 4).

Regarding the degree of case complexity, most of the respondent dental schools in the different geographical areas allow undergraduate students to treat uncomplicated cases with low difficulty (based on the AAE case difficulty assessment form). Additionally, cases with moderate difficulty are also allowed by most respondent dental schools, except in Asia and North America (Table 4).

### Assessment methods

The majority of participating schools employed written exams with free text and multiple-choice questions (MCQs) (Table 5). In terms of the practical component, the majority of dental schools evaluate the students using a real clinical setting (on patients). The Objective Structured Clinical Examination (OSCE) was used in more than half of Australian and African schools. Portfolio was utilized in more than half of the schools in North America and Australia. Students were requested to present their clinical work in over 50% of the schools in South America and Africa (Table 5).

**Table 5:** Assessment methods used in endodontic courses (n, %).

	Asia (*n* = 12)	Africa (*n* = 7)	Europe (*n* = 7)	Australia (*n* = 6)	North America (*n* = 3)	South America (*n* = 3)	Total (*n* = 38)
Theoretical part							
Multiple choice (MCQs)	11 (91.7%)	6 (85.7%)	5 (71.4%)	5 (83.3%)	3 (100.0%)	3 (100.0%)	33 (86.8%)
Written exam with free text	8 (66.7%)	5 (71.4%)	5 (71.4%)	6 (100.0%)	1 (33.3%)	2 (66.7%)	27 (71.1%)
Oral examination	7 (58.3%)	2 (28.6%)	3 (42.9%)	1 (16.7%)	1 (33.3%)	2 (66.7%)	16 (42.1%)
Objective structured Oral examination OSOE	3 (25.0%)	1 (14.3%)	2 (28.6%)	2 (33.3%)	0 (0.0%)	0 (0.0%)	8 (21.1%)
Other:	0 (0.0%)	0 (0.0%)	0 (0.0%)	0 (0.0%)	0 (0.0%)	0 (0.0%)	0 (0.0%)
**Practical part**							
On patients	10 (83.3%)	5 (71.4%)	4 (57.1%)	3 (50.0%)	2 (66.7%)	2 (66.7%)	26 (68.4%)
OSCE	4 (33.3%)	4 (57.1%)	1 (14.3%)	3 (50.0%)	1 (33.3%)	1 (33.3%)	14 (36.8%)
In an alternative (e.g., Mini-CEX = Mini-CT Evaluation Exercise, DOPS = Direct Observation of Procedural Skills)	2 (16.7%)	2 (28.6%)	3 (42.9%)	1 (16.7%)	0 (0.0%)	0 (0.0%)	8 (21.1%)
On human extracted teeth	5 (41.7%)	3 (42.9%)	3 (42.9%)	0 (0.0%)	0 (0.0%)	2 (66.7%)	13 (34.2%)
On industrially produced standardized simulation teeth made of plastic	1 (8.3%)	0 (0.0%)	2 (28.6%)	2 (33.3%)	0 (0.0%)	0 (0.0%)	5 (13.2%)
Portfolio	2 (16.7%)	1 (14.3%)	1 (14.3%)	3 (50.0%)	2 (66.7%)	0 (0.0%)	9 (23.7%)
Case presentation of CT work	5 (41.7%)	4 (57.1%)	1 (14.3%)	0 (0.0%)	0 (0.0%)	2 (66.7%)	12 (31.6%)
Other:	0 (0.0%)	0 (0.0%)	1 (14.3%)	0 (0.0%)	0 (0.0%)	0 (0.0%)	1 (2.6%)

## Discussion

Considering variations among dental institutions in terms of location, staff, enrolment, and financial resources, understanding the endodontic curriculum is crucial. A solid foundation in Endodontology is vital for future dentists and should be established during undergraduate education. Previous research has been conducted in various countries^13–18^, providing some insights into endodontic education in the European and Asian regions.

This study provides the first global overview of undergraduate endodontic teaching, encompassing data from 38 dental schools across six continents.

Previous studies did not reveal whether the pre-clinical course is taught as a separate course or integrated with other courses. The present study showed that most of the dental schools (63.2%) provide the preclinical course as a separate course, except the 3 participating South American schools. Some European endodontic programs opt for comprehensive clinic settings rather than specialized clinical training. The Malaysian National Survey (2020) noted that collaboration with other disciplines in endodontic education—particularly during seminars, problem-based learning (PBL), and case presentations—helps students understand patient management from various perspectives, promoting holistic patient care^17^. However, it was unclear whether this integrated approach is applied in PT or CT since the responding schools reported a focus on didactic endodontic instruction.

Traditional phantom heads using artificial and extracted teeth, which are mounted as models on mannequins, are generally used for practicing irreversible dental procedures. However, acquiring extracted teeth is becoming increasingly challenging, and the sensory feedback from preparing plastic teeth differs significantly from that of natural teeth^19^. Traditional manikins are still utilized for practical instruction across all continents; the use of virtual reality simulators is still limited to 15%. Three schools in Europe, Australia, and Africa lacked dental simulators. Extracted human teeth are used in 78.9% of schools, and 42.1% use commercial plastic teeth, predominantly in Australia and North America. This aligns with previous studies^13–16^showing that students mostly perform their PT on natural extracted teeth. Despite the high cost of virtual reality simulators, they offer an affordable alternative to costly artificial teeth with modified root canal systems with the opportunity to practice repeatedly^20^. However, limited budgets and concerns about their training effectiveness may hinder its broader adoption^21^.

Endodontic topics are taught over one or more academic years, with the timing varying by dental schools. African and Asian schools offer PT later than other dental schools participating in this study. This is due to the variation in the duration of the dentistry undergraduate program, which varies in different geographical areas from four to six years.

Early coverage of endodontic topics in the curriculum of undergraduate dental programs would give the students the chance to be taught a wide range of topics, which would help in establishing a good foundation of knowledge; that can be reflected later in the practical postgraduate performance.

Clinical instruction is typically delayed until the fourth and fifth years in Asia and Africa, while in the other continents, it generally occurs earlier in the third and fourth years. For example, 40% of UK schools include Endodontics instruction in the second and third years^13^, 95% of Spanish dental schools offer it in the fourth year^14^, and in Italy, instruction is focused on the third and fourth years^16^, Alobaid et al. (2022) reported that 66% of the colleges in Saudi Arabia prefer to teach theoretical knowledge of the advanced endodontic topics in the internship year, and general basic endodontic topics are mainly covered starting from year 2^15^.

According to ESE guidelines, schools have the flexibility to determine the number of hours allocated for theoretical and practical endodontic instruction^8^. On average, 52.5 h are dedicated to teaching endodontics theory, with a range from 15 to 90 h. In North American schools, the minimum requirement is 90 h. While half of Australian and Asian dental schools assign 45 to 90 h for theoretical endodontics, time is varying in the other continents. The variation in time allocated for teaching endodontics theory may be attributed to the misunderstandings of the questionnaire questions, which were same reported in other previous studies^13,18^.

Both European and Australian dental schools showed a higher percentage for students’ enrolment than other continents, which will have a reflection on staff: student ratio and the educational outcomes.

Effective endodontic training depends on the ESE-recommended staff: student ratios^8^. Safe practice, guidance of students throughout the learning process, and close interaction would be ensured if staff: students’ ratio is high^13^. ESE recommended that “six endodontic treatments are normally considered the maximum that a staff member should supervise simultaneously. For CT, however, a maximum of four is recommended” by the ESE^3^. Considering this ESE recommendations for staff: student ratio, results from Asia, Europe, and Australia showed more favorable ratios in CT of 1:6 staff: student ratio, while all other respondents are exceeding the recommended staff: student ratio. The variations in ratios may be due to differences in resource allocation and the level of emphasis placed on endodontic education. These results are comparable to previous reports in comprehensive surveys^13–16^except in German-speaking nations, where Sacha et al.(2021) reported a ratio in preclinical courses ranging from 1:4 to 1:38, with an average of 1:15. However, the updated German licensing regulations for dentists changed the faculty-to-student ratio for the phantom head course from 1:20 to 1:15. starting in October 2020^18^.

The ESE emphasized supervising undergraduate students by qualified Endodontists or dentists who have specific training in Endodontics to positively impact the educational outcomes^3^. In most geographical areas, PC and CT are supervised by endodontists. This shift can be seen as a significant improvement. However, in South America, GDPs often take on this role, which could be due to the lack of formal endodontic specialty programs in some regions. On the other hand, postgraduate endodontics students from institutions with postgraduate programs can function as teaching assistants for undergraduate preclinical and clinical endodontics courses, as GDPs with a special interest in endodontics. This might explain why more than half of African dental schools GDPs with particular endodontic interests are contributors for endodontic training for undergraduate dental programs^14^.

In the current study, the schools in various locations provide both practical classes and didactic lectures. In Africa, Europe, Australia, and South America, seminars are more prevalent than in Asia and North America. Australian and African schools frequently employ problem-based learning(PBL). This finding complies with the previous studies which showed that dental schools generally use combinations of teaching methods, with the highest percentage is for lectures and seminars^13–18^. In Italy, problem-based learning varies from its implementation in England and Spain^13,14,16^. Alobaid et al.(2022) showed that virtual simulation and reading lists are the least preferred methods of learning in Saudi dental schools^15^. According to Nagendrababu et al. (2018)^22^, the systematic review found no significant difference in knowledge gain and performance between technology-enhanced learning and traditional methods for Endodontic instruction for dental students. Incorporating Gamification and Game-Based Learning (GBL) as complementary tool in traditional classroom setting can enhance dental education by improving knowledge retention, student engagement, and overall satisfaction among both graduate and postgraduate students^23–26^. Additionally, Artificial Intelligence (AI) is transforming dental education by offering tools that enhance diagnostic accuracy, assist in treatment planning, and support the development of clinical decision-making skills^27^. However, our study did not assess the extent to which these methods have been integrated into undergraduate endodontic curricula. Future research should evaluate the impact of these technologies on knowledge acquisition, clinical performance, and soft skills development in endodontics.

The ESE guidelines set the scope and essentials of endodontic instruction^3^. Our study explored various aspects of the ESE endodontic curriculum; the findings indicate that many schools cover these topics comprehensively. However, orthodontic concerns are frequently overlooked in the curriculum.

Based on the results of this study, it was commonly observed across all participating dental schools that students primarily engage in non-surgical RCT and vital pulp therapy. Only a minority of schools reported including non-surgical root canal retreatments in their CT. These findings align with previous studies. While this study corroborates earlier research regarding the lower percentage of students performing bleaching of endodontically treated teeth, all dental schools in North America reported that their students perform such procedures. Additionally, 66.7% of Australian dental schools included bleaching of endodontically treated teeth in their training, a higher percentage compared to a previous study^14^.

Performing more complex treatments on patients during the early stages of clinical experience is not advisable, as it can negatively affect student self-efficacy^28^. Previous study showed found that 80% of students felt anxious about managing molars and time during RCTs, and 47% of novice students pursued professional development courses shortly after graduation to seek career growth opportunities^29^. In this study, Friedlander et al. (2023), recommended maximum clinical experience under the supervision of endodontic teachers is crucial for boosting confidence and alleviating anxiety^29^.

Talking about surgical endodontic treatment, only one European dental school provides their students training for it. This is a common result in previous studies^13,30^. However, the ESE recommended that undergraduate dental students need only to be familiar with such procedures by direct assistance or observation^8^.

A minimum number of RCT cases was mandatory for most schools during PT and CT training. Evaluating student performance consistently to a high standard is more essential than dealing with a large number of cases. Students should master the skills required for simple root canal procedures on both anterior and posterior teeth^31^. The ESE undergraduate guidelines^3,31^place more emphasis on measuring the quality and consistency of student performance than on specifying a particular minimum number of procedures^3^.

In contemporary dental schools, while some advanced endodontic technologies like electronic apex locators, new rotary instruments, and new bioceramic cements have been adopted, the extensive use of modern root filling techniques, magnification, and ultrasonic instruments remains limited.

In endodontics, the use of loupes is the new minimum standard for vision, and undergraduate teaching^32^enhancing both visual acuity and ergonomic, making them an essential tool in PT and CT. In the current study, over 50% respondents in Asia and Africa do not use magnification systems during PT or CT, compared to North America, Australia, and Europe, which is similar to previous reports^13–15^, while 82% of dental schools in German-speaking nations offered PT with access to dental microscopes^18^. According to Mergoni et al. (2022)^16^, magnifying systems were used in 35.7% of schools during PT, and the microscope was used in 21.4% of schools. Insufficient staff training and high costs could be the barriers of teaching undergraduate endodontics with magnification^32^. The use of magnification in endodontic training needs to be added to the curriculum in African and Asian dental schools as to keep the students updated with recent advances in endodontic treatments.

Ultrasonic instruments are seldom used in undergraduate education. 94.7% and 76.3% of schools in all areas failed to implement ultrasound for PT and CT. Comparable results were observed in previous studies^14–16,18^. In the UK, 53% of schools employed ultrasonic devices for access cavity preparation and irrigant agitation during PT, while 20% did not implement them clinical^13^. Dental schools should prioritize enhancing students’ familiarity with ultrasound devices in preclinical settings.

In comparison to ultrasonic use, apex locators as a mean for determining working length are incorporated in all dental schools, either for CT or many for PT.

Most dental schools teach their students to perform RCT using both manual and engine-driven NiTi files^13–17^. This reflects the shift of educational institutions towards adopting advanced technologies. However, students still utilize manual instruments to develop the skills needed when assigned to rural dental clinics, where access to endodontic instruments may be restricted^17^.

Similar to previous reports^15,17^, the step-back technique is the most common manual technique used in more than half of dental schools.

Regarding endodontic irrigation, the results of the current study are consistent with those of others, where NaOCl is the most commonly used irrigant^13–16^.

As in previous studies^15,17^, calcium hydroxide continues to be the most common intracanal medicament in multiple visit treatments.

Despite the availability of various obturation techniques, cold lateral compaction is the most commonly taught method of obturation (except in North America), followed by single cone obturation. This finding is consistent with previous studies^13,14,17^. Cold lateral condensation is remains the common technique due to its adaptability, affordability, and simplicity^33^.

Advanced endodontic technologies employ MTA as the preferred material, surpassing CBCT and thermally treated Niti files. Most dental schools in UK used MTA, Biodentine and BioRoot in their CT^13^. However, this study showed lower percentages for the use of MTA, Biodentine, or Bioceramic sealers in CT, which could be directly related to cost issues^17^. Only half of the schools reported placing definitive restoration after completion of RCT in CT, this aspect should be encouraged to ensure good seal and maximize treatment outcome.

Establishing a case’s level of difficulty is crucial for minimizing procedural errors^34^and enabling practitioners to manage RCT treatments safely within their scope while recognizing the need for referrals. New graduates should be proficient in delivering patient care, cognizant of their boundaries in practice, competency, and limitations, and willing to refer cases surpassing their expertise^3,34^.

More than half of clinical case assignments for undergraduate students in Australia and North America adhered to the American Association of Endodontics difficulty classification assessment. In Asia (67.7%), Africa (42.9%), Europe (71.4%), and South America (100%), no specialized assessments were applied for evaluating school cases based on complexity. A fact that requires attention.

In the current study, over 50% of schools in South America and Africa enforce a requirement for students to present their clinical work, enhancing their capacity to apply theoretical knowledge in real-life situations and boosting critical thinking skills.

The study reveals that dental schools use various assessment methods, including free text questions, multiple-choice questions, and portfolios. Educators should evaluate student performance in endodontic procedures to ensure competence. Formative assessments can monitor progress and provide feedback. Competency demonstration in clinical endodontic management and case review is recommended^35^. The current study complies with this recommendation, with 68.4% of dental schools requiring practical assessments on patients and 31.6% mandating case presentations.

Incorporating modern, up-to-date technologies and techniques into undergraduate endodontic education is essential to adequately prepare students for clinical practice. The study revealed the need for improvements in the undergraduate endodontic curriculum in terms of student-to-staff ratios, teaching and assessment methods, and incorporation of modern techniques.

The study revealed that while fundamental knowledge is being established in some countries, curricular reform is a global trend. Collaboration between educators and policymakers is crucial for updating curricula in response to endodontic advancements, requiring investments in facilities, human resources, and research. Educational standards should promote excellence and guide teachers through data analysis to enhance instruction. This paper may encourage cooperation among diverse dental schools in the future. The impact of financial resources on endodontic education warrants further investigation. Harmonization of undergraduate endodontic education globally could help ensure higher quality and standardized dental care delivery by general dental practitioners worldwide, though further research is needed to identify the most effective strategies for achieving this goal.

### Limitations of the study

Recognizing the study’s limitations is crucial for interpreting its impact. While this research provides valuable insights into global endodontic education, some constraints should be noted.

Future studies should strive for a more balanced and representative sample across continents, particularly by including populous countries with significant demographic variability, such as China, Brazil, and Russia. Addressing the underrepresentation of North and South America and the overrepresentation of Asia will help mitigate regional bias and enhance the generalizability of findings. Furthermore, future research should conduct separate, in-depth analyses of preclinical and clinical endodontic curricula to provide a more comprehensive understanding of global trends. Due to ethical considerations and confidentiality agreements, specific university details and visual representations are not included. Future studies should explore ways to enhance transparency while maintaining confidentiality Another key area for further investigation is the collection of data on sodium hypochlorite concentration, which was not included in this study.

## Conclusion

Incorporating more modern technologies, implementing different teaching and assessment strategies, and enhancing the student: staff ratios are fundamentals need to be taken into consideration by the educational institutions when reforming the undergraduate dental curriculum. Harmonization of undergraduate endodontic education globally could help ensure higher quality and standardized dental care delivery by GDPs all over the world.

### Methods and materials

Ethical approval was obtained from the Institution Research Board Committee. Dental faculty members who teach undergraduate endodontics at fifty dental institutions across six continents were invited to participate in an online survey that was created using a web-based survey tool (Google Form). The study design was a cross-sectional online survey, which was conducted as a global pilot study. Dental faculty were recruited using a convenience sampling technique. Invitations to take part in the study were distributed by email, social media, and individual contact lists at the potential universities. Data collection involved modifying a previously validated questionnaire by Sacha et al., 2021 16. The survey comprised of 46 close-ended questions in 3 sections on essential aspects of the didactic, PT, CT, and assessments in undergraduate endodontic programs (Appendix 1). The majority of questions were multiple-choice, though occasionally there is more than one viable response (indicated by a strike). Additionally, some questions allowed participants to enter free-text answers. Each participant had to read an information sheet that outlined the goal and scope of the study, and to electronically sign a consent form before they could access the questionnaire. Responses were collected between June 1, 2023, and September 1, 2023. Descriptive analysis was employed for each question using SPSS 29 (IBM SPSS Inc., Chicago, IL, USA).

## Electronic supplementary material

Below is the link to the electronic supplementary material.

Supplementary Material 1

## Data Availability

Data is available from the corresponding author upon reasonable request.
