# Supplementary material for: Global trends in preclinical and clinical undergraduate endodontic education: A worldwide survey
Source: Sci Rep. 2025 Mar 24;15:10078. doi: 10.1038/s41598-025-94836-y (PMC11933302; doi:10.1038/s41598-025-94836-y)
Supplement: Supplementary file 1 — Supplementary Material 1 [file 41598_2025_94836_MOESM1_ESM.docx]

**Supplementary tables:**

**Table S1 In what year/years are preclinical and clinical endodontics taught? (n, %)**

|  | **Asia (n = 12)** | | **Africa (n = 7)** | | **Europe (n = 7)** | | **Australia (n = 6)** | | **North America (n = 3)** | | **South America (n = 3)** | | **Total (n = 38)** | |
| --- | --- | --- | --- | --- | --- | --- | --- | --- | --- | --- | --- | --- | --- | --- |
| **Year** | **PT** | **CT** | **PT** | **CT** | **PT** | **CT** | **PT** | **CT** | **PT** | **CT** | **PT** | **CT** | **PT** | **CT** |
| First year | 2 (16.7%) | 0 (0.0%) | 0 (0.0%) | 0 (0.0%) | 1 (14.3%) | 0 (0.0%) | 0 (0.0%) | 0 (0.0%) | 0 (0.0%) | 0 (0.0%) | 1 (33.3%) | 0 (0.0%) | 4 (10.5%) | 0 (0.0%) |
| Second year | 4 (33.3%) | 0 (0.0%) | 1 (14.3%) | 0 (0.0%) | 4 (57.1%) | 0 (0.0%) | 3 (50.0%) | 0 (0.0%) | 3 (100.0%) | 0 (0.0%) | 2 (66.7%) | 1 (33.3%) | 17 (44.7%) | 1 (2.6%) |
| Third year | 4 (33.3%) | 4 (33.3%) | 5 (71.4%) | 0 (0.0%) | 4 (57.1%) | 4 (57.1%) | 3 (50.0%) | 5 (83.3%) | 0 (0.0%) | 3 (100.0%) | 1 (33.3%) | 2 (66.7%) | 17 (44.7%) | 18 (47.4%) |
| Fourth year | 7 (58.3%) | 8 (66.7%) | 3 (42.9%) | 6 (85.7%) | 0 (0.0%) | 6 (85.7%) | 1 (16.7%) | 2 (33.3%) | 0 (0.0%) | 3 (100.0%) | 0 (0.0%) | 2 (66.7%) | 11 (28.9%) | 27 (71.1%) |
| Fifth year | 0 (0.0%) | 11 (91.7%) | 0 (0.0%) | 6 (85.7%) | 0 (0.0%) | 4 (57.1%) | 0 (0.0%) | 1 (16.7%) | 0 (0.0%) | 0 (0.0%) | 0 (0.0%) | 1 (33.3%) | 0 (0.0%) | 23 (60.5) |
| Sixth year | 0 (0.0%) | 4 (33.3%) | 0 (0.0%) | 1 (14.3%) | 0 (0.0%) | 0 (0.0%) | 0 (0.0%) | 0 (0.0%) | 0 (0.0%) | 0 (0.0%) | 0 (0.0%) | 0 (0.0%) | 0 (0.0%) | 5 (13.2%) |

**PT: Preclinical training, CT: Clinical training**

**Table S2 Total hours (1hour = 45 minutes) allocated to the theoretical part of endodontic training and number of the students attending endodontic courses (n, %)**

|  | **Asia (n = 12)** | **Africa (n = 7)** | **Europe (n = 7)** | **Australia (n = 6)** | **North America (n = 3)** | **South America (n = 3)** | **Total (n = 38)** |
| --- | --- | --- | --- | --- | --- | --- | --- |
| **Total hours (1hour = 45 minutes) allocated to the theoretical part of endodontic training** | | | | | | | |
| 15 hours | 0 (0.0%) | 1 (14.3%) | 0 (0.0%) | 1 (16.7%) | 0 (0.0%) | 0 (0.0%) | 2 (5.3%) |
| 30 hours | 1 (8.3%) | 1 (14.3%) | 0 (0.0%) | 2 (33.3%) | 0 (0.0%) | 0 (0.0%) | 4 (10.5%) |
| 45 hours | 2 (16.7%) | 1 (14.3%) | 2 (28.6%) | 3 (50.0%) | 0 (0.0%) | 0 (0.0%) | 8 (21.1%) |
| 60 hours | 2 (16.7%) | 2 (28.6%) | 2 (28.6%) | 0 (0.0%) | 0 (0.0%) | 1 (33.3%) | 7 (18.4%) |
| 75 hours | 0 (0.0%) | 1 (14.3%) | 0 (0.0%) | 0 (0.0%) | 0 (0.0%) | 1 (33.3%) | 2 (5.3%) |
| 90 hours | 6 (50.0%) | 0 (0.0%) | 2 (28.6%) | 0 (0.0%) | 3 (100.0%) | 1 (33.3%) | 12 (31.6%) |
| Other: (81) | 0 (0.0%) | 1 (14.3%) | 0 (0.0%) | 0 (0.0%) | 0 (0.0%) | 0 (0.0%) | 1 (2.6%) |
| Other: (135) | 1 (8.3%) | 0 (0.0%) | 0 (0.0%) | 0 (0.0%) | 0 (0.0%) | 0 (0.0%) | 1 (2.6%) |
| Other: (200) | 0 (0.0%) | 0 (0.0%) | 1 (14.3%) | 0 (0.0%) | 0 (0.0%) | 0 (0.0%) | 1 (2.6%) |
|  | | | | | | | |
| **Number of the students attending endodontic courses** | | | | | | | |
| ≤14 | 1 (8.3%) | 0 (0.0%) | 1 (14.3%) | 0 (0.0%) | 0 (0.0%) | 1 (33.3%) | 3 (7.9%) |
| 15 – 29 | 0 (0.0%) | 0 (0.0%) | 0 (0.0%) | 0 (0.0%) | 0 (0.0%) | 1 (33.3%) | 1 (2.6%) |
| 30 – 44 | 2 (16.7%) | 1 (14.3%) | 2 (28.6%) | 1 (16.7%) | 0 (0.0%) | 0 (0.0%) | 6 (15.8%) |
| 45 – 59 | 3 (25.0%) | 1 (14.3%) | 1 (14.3%) | 0 (0.0%) | 0 (0.0%) | 1 (33.3%) | 6 (15.8%) |
| 60 – 74 | 0 (0.0%) | 2 (28.6%) | 0 (0.0%) | 1 (16.7%) | 1 (33.3%) | 0 (0.0%) | 4 (10.5%) |
| 75 – 89 | 1 (8.3%) | 0 (0.0%) | 0 (0.0%) | 1 (16.7%) | 1 (33.3%) | 0 (0.0%) | 3 (7.9%) |
| 90 – 104 | 3 (25.0%) | 1 (14.3%) | 0 (0.0%) | 3 (50.0%) | 0 (0.0%) | 0 (0.0%) | 7 (18.4%) |
| More than 104 | 2 (16.7%) | 2 (28.6%) | 3 (42.9%) | 0 (0.0%) | 1 (33.3%) | 0 (0.0%) | 8 (21.1%) |

**Table S3 Teaching strategies and subjects taught in each year (n, %)**

|  | **Asia (n = 12)** | **Africa (n = 7)** | **Europe (n = 7)** | **Australia (n = 6)** | **North America (n = 3)** | **South America (n = 3)** | **Total (n = 38)** |
| --- | --- | --- | --- | --- | --- | --- | --- |
| **Teaching strategies** | | | | | | | |
| Lectures | 11 (91.7%) | 6 (85.7%) | 7 (100.0%) | 6 (100.0%) | 3 (100.0%) | 2 (66.7%) | 35 (92.1%) |
| Seminars (mostly interdisciplinary discussion of content in smaller groups than in the lectures) | 5 (41.7%) | 5 (71.4%) | 4 (57.1%) | 5 (83.3%) | 1 (33.3%) | 2 (66.7%) | 22 (57.9%) |
| Tutorials= (are led by students with appropriate qualiﬁcations, supervised by members of the teaching staff and represent | 3 (25.0%) | 2 (28.6%) | 5 (71.4%) | 1 (16.7%) | 0 (0.0%) | 2 (66.7%) | 13 (34.2%) |
| Problem based learning | 3 (25.0%) | 3 (42.9%) | 3 (42.9%) | 3 (50.0%) | 1 (33.3%) | 2 (66.7%) | 15 (39.5%) |
| Independent study | 1 (8.3%) | 1 (14.3%) | 2 (28.6%) | 3 (50.0%) | 2 (66.7%) | 1 (33.3%) | 10 (26.3%) |
| Projects/ assignments | 1 (8.3%) | 1 (14.3%) | 2 (28.6%) | 2 (33.3%) | 1 (33.3%) | 1 (33.3%) | 8 (21.1%) |
| E-learning | 4 (33.3%) | 3 (42.9%) | 2 (28.6%) | 2 (33.3%) | 2 (66.7%) | 1 (33.3%) | 14 (36.8%) |
| Laboratory and practical learning | 11 (91.7%) | 4 (57.1%) | 5 (71.4%) | 6 (100.0%) | 2 (66.7%) | 2 (66.7%) | 30 (78.9%) |
| Videos | 6 (50.0%) | 5 (71.4%) | 4 (57.1%) | 5 (83.3%) | 3 (100.0%) | 0 (0.0%) | 23 (60.5%) |
| Manuals | 4 (33.3%) | 3 (42.9%) | 4 (57.1%) | 5 (83.3%) | 2 (66.7%) | 0 (0.0%) | 18 (47.4%) |
| Reading lists | 6 (50.0%) | 1 (14.3%) | 3 (42.9%) | 2 (33.3%) | 1 (33.3%) | 2 (66.7%) | 15 (39.5%) |
| Other | 1 (8.3%) | 0 (0.0%) | 0 (0.0%) | 1 (16.7%) | 1 (33.3%) | 0 (0.0%) | 3 (7.9%) |
|  |  |  |  |  |  |  |  |
| **Topics** | | | | | | | |
| **Root canal anatomy and pulp histology** |  |  |  |  |  |  |  |
| Non | 0 (0.0%) | 0 (0.0%) | 0 (0.0%) | 0 (0.0%) | 0 (0.0%) | 0 (0.0%) | 0 (0.0%) |
| First year | 3 (25.0%) | 2 (28.6%) | 2 (28.6%) | 3 (50.0%) | 0 (0.0%) | 2 (66.7%) | 12 (31.6%) |
| Second year | 4 (33.3%) | 2 (28.6%) | 3 (42.9%) | 2 (33.3%) | 3 (100.0%) | 1 (33.3%) | 15 (39.5%) |
| Third year | 5 (41.7%) | 5 (71.4%) | 2 (28.6%) | 1 (16.7%) | 1 (33.3%) | 0 (0.0%) | 14 (36.8%) |
| Fourth year | 5 (41.7%) | 1 (14.3%) | 0 (0.0%) | 0 (0.0%) | 1 (33.3%) | 0 (0.0%) | 7 (18.4%) |
| Fifth year | 1 (8.3%) | 0 (0.0%) | 0 (0.0%) | 0 (0.0%) | 0 (0.0%) | 0 (0.0%) | 1 (2.6%) |
| Sixth year | 0 (0.0%) | 0 (0.0%) | 0 (0.0%) | 0 (0.0%) | 0 (0.0%) | 0 (0.0%) | 0 (0.0%) |
|  |  |  |  |  |  |  |  |
| **Pulp physiopathology** |  |  |  |  |  |  |  |
| Non | 0 (0.0%) | 0 (0.0%) | 0 (0.0%) | 0 (0.0%) | 0 (0.0%) | 0 (0.0%) | 0 (0.0%) |
| First year | 2 (16.7%) | 1 (14.3%) | 1 (14.3%) | 3 (50.0%) | 1 (33.3%) | 1 (33.3%) | 9 (23.7%) |
| Second year | 5 (41.7%) | 1 (14.3%) | 3 (42.9%) | 2 (33.3%) | 2 (66.7%) | 2 (66.7%) | 15 (39.5%) |
| Third year | 4 (33.3%) | 4 (57.1%) | 1 (14.3%) | 1 (16.7%) | 0 (0.0%) | 1 (33.3%) | 11 (28.9%) |
| Fourth year | 4 (33.3%) | 1 (14.3%) | 2 (28.6%) | 0 (0.0%) | 0 (0.0%) | 0 (0.0%) | 7 (18.4%) |
| Fifth year | 1 (8.3%) | 0 (0.0%) | 0 (0.0%) | 0 (0.0%) | 0 (0.0%) | 0 (0.0%) | 1 (2.6%) |
| Sixth year | 0 (0.0%) | 0 (0.0%) | 0 (0.0%) | 0 (0.0%) | 0 (0.0%) | 0 (0.0%) | 0 (0.0%) |
|  |  |  |  |  |  |  |  |
| **Endodontic microbiology** |  |  |  |  |  |  |  |
| Non | 0 (0.0%) | 0 (0.0%) | 0 (0.0%) | 0 (0.0%) | 0 (0.0%) | 0 (0.0%) | 0 (0.0%) |
| First year | 1 (8.3%) | 1 (14.3%) | 0 (0.0%) | 2 (33.3%) | 0 (0.0%) | 1 (33.3%) | 5 (13.2%) |
| Second year | 3 (25.0%) | 1 (14.3%) | 3 (42.9%) | 3 (50.0%) | 3 (100.0%) | 1 (33.3%) | 14 (36.8%) |
| Third year | 6 (50.0%) | 4 (57.1%) | 2 (28.6%) | 2 (33.3%) | 0 (0.0%) | 1 (33.3%) | 15 (39.5%) |
| Fourth year | 5 (41.7%) | 1 (14.3%) | 2 (28.6%) | 0 (0.0%) | 0 (0.0%) | 0 (0.0%) | 8 (21.1%) |
| Fifth year | 1 (8.3%) | 0 (0.0%) | 0 (0.0%) | 0 (0.0%) | 0 (0.0%) | 0 (0.0%) | 1 (2.6%) |
| Sixth year | 0 (0.0%) | 0 (0.0%) | 0 (0.0%) | 0 (0.0%) | 0 (0.0%) | 0 (0.0%) | 0 (0.0%) |
|  |  |  |  |  |  |  |  |
| **Endodontic radiology** |  |  |  |  |  |  |  |
| Non | 0 (0.0%) | 0 (0.0%) | 0 (0.0%) | 0 (0.0%) | 0 (0.0%) | 0 (0.0%) | 0 (0.0%) |
| First year | 2 (16.7%) | 1 (14.3%) | 0 (0.0%) | 1 (16.7%) | 0 (0.0%) | 1 (33.3%) | 5 (13.2%) |
| Second year | 1 (8.3%) | 0 (0.0%) | 4 (57.1%) | 4 (66.7%) | 3 (100.0%) | 1 (33.3%) | 13 (34.2%) |
| Third year | 7 (58.3%) | 5 (71.4%) | 4 (57.1%) | 3 (50.0%) | 0 (0.0%) | 1 (33.3%) | 20 (52.6%) |
| Fourth year | 4 (33.3%) | 1 (14.3%) | 2 (28.6%) | 0 (0.0%) | 0 (0.0%) | 0 (0.0%) | 7 (18.4%) |
| Fifth year | 1 (8.3%) | 0 (0.0%) | 2 (28.6%) | 0 (0.0%) | 0 (0.0%) | 0 (0.0%) | 3 (7.9%) |
| Sixth year | 0 (0.0%) | 0 (0.0%) | 0 (0.0%) | 0 (0.0%) | 0 (0.0%) | 0 (0.0%) | 0 (0.0%) |
|  |  |  |  |  |  |  |  |
| **Endodontic materials** |  |  |  |  |  |  |  |
| Non | 0 (0.0%) | 0 (0.0%) | 0 (0.0%) | 0 (0.0%) | 0 (0.0%) | 0 (0.0%) | 0 (0.0%) |
| First year | 3 (25.0%) | 0 (0.0%) | 0 (0.0%) | 0 (0.0%) | 0 (0.0%) | 1 (33.3%) | 4 (10.5%) |
| Second year | 4 (33.3%) | 1 (14.3%) | 3 (42.9%) | 5 (83.3%) | 3 (100.0%) | 1 (33.3%) | 17 (44.7%) |
| Third year | 4 (33.3%) | 5 (71.4%) | 6 (85.7%) | 2 (33.3%) | 0 (0.0%) | 1 (33.3%) | 18 (47.4%) |
| Fourth year | 5 (41.7%) | 2 (28.6%) | 2 (28.6%) | 0 (0.0%) | 0 (0.0%) | 0 (0.0%) | 9 (23.7%) |
| Fifth year | 1 (8.3%) | 1 (14.3%) | 2 (28.6%) | 0 (0.0%) | 0 (0.0%) | 0 (0.0%) | 4 (10.5%) |
| Sixth year | 0 (0.0%) | 0 (0.0%) | 0 (0.0%) | 0 (0.0%) | 0 (0.0%) | 0 (0.0%) | 0 (0.0%) |
|  |  |  |  |  |  |  |  |
| **Vital pulp therapies** |  |  |  |  |  |  |  |
| Non | 0 (0.0%) | 0 (0.0%) | 0 (0.0%) | 0 (0.0%) | 0 (0.0%) | 0 (0.0%) | 0 (0.0%) |
| First year | 1 (8.3%) | 0 (0.0%) | 0 (0.0%) | 1 (16.7%) | 0 (0.0%) | 1 (33.3%) | 3 (7.9%) |
| Second year | 2 (16.7%) | 1 (14.3%) | 3 (42.9%) | 2 (33.3%) | 3 (100.0%) | 0 (0.0%) | 11 (28.9%) |
| Third year | 2 (16.7%) | 1 (14.3%) | 3 (42.9%) | 3 (50.0%) | 1 (33.3%) | 2 (66.7%) | 12 (31.6%) |
| Fourth year | 8 (66.7%) | 3 (42.9%) | 4 (57.1%) | 2 (33.3%) | 2 (66.7%) | 0 (0.0%) | 19 (50.0%) |
| Fifth year | 4 (33.3%) | 3 (42.9%) | 2 (28.6%) | 0 (0.0%) | 0 (0.0%) | 0 (0.0%) | 9 (23.7%) |
| Sixth year | 0 (0.0%) | 0 (0.0%) | 0 (0.0%) | 0 (0.0%) | 0 (0.0%) | 0 (0.0%) | 0 (0.0%) |
|  |  |  |  |  |  |  |  |
| **Root canal treatment of immature teeth** |  |  |  |  |  |  |  |
| Non | 1 (8.3%) | 0 (0.0%) | 0 (0.0%) | 0 (0.0%) | 0 (0.0%) | 0 (0.0%) | 1 (2.6%) |
| First year | 0 (0.0%) | 0 (0.0%) | 0 (0.0%) | 0 (0.0%) | 0 (0.0%) | 1 (33.3%) | 1 (2.6%) |
| Second year | 2 (16.7%) | 1 (14.3%) | 0 (0.0%) | 2 (33.3%) | 2 (66.7%) | 0 (0.0%) | 7 (18.4%) |
| Third year | 2 (16.7%) | 0 (0.0%) | 3 (42.9%) | 4 (66.7%) | 0 (0.0%) | 0 (0.0%) | 9 (23.7%) |
| Fourth year | 8 (66.7%) | 5 (71.4%) | 5 (71.4%) | 1 (16.7%) | 1 (33.3%) | 2 (66.7%) | 22 (57.9%) |
| Fifth year | 4 (33.3%) | 4 (57.1%) | 2 (28.6%) | 0 (0.0%) | 0 (0.0%) | 0 (0.0%) | 10 (26.3%) |
| Sixth year | 0 (0.0%) | 0 (0.0%) | 0 (0.0%) | 0 (0.0%) | 0 (0.0%) | 0 (0.0%) | 0 (0.0%) |
|  |  |  |  |  |  |  |  |
| **Non-surgical endodontic treatment** |  |  |  |  |  |  |  |
| Non | 0 (0.0%) | 0 (0.0%) | 0 (0.0%) | 0 (0.0%) | 0 (0.0%) | 0 (0.0%) | 0 (0.0%) |
| First year | 0 (0.0%) | 0 (0.0%) | 0 (0.0%) | 0 (0.0%) | 0 (0.0%) | 1 (33.3%) | 1 (2.6%) |
| Second year | 3 (25.0%) | 2 (28.6%) | 3 (42.9%) | 4 (66.7%) | 3 (100.0%) | 0 (0.0%) | 15 (39.5%) |
| Third year | 3 (25.0%) | 3 (42.9%) | 5 (71.4%) | 4 (66.7%) | 1 (33.3%) | 1 (33.3%) | 17 (44.7%) |
| Fourth year | 7 (58.3%) | 6 (85.7%) | 2 (28.6%) | 0 (0.0%) | 1 (33.3%) | 1 (33.3%) | 17 (44.7%) |
| Fifth year | 4 (33.3%) | 1 (14.3%) | 0 (0.0%) | 0 (0.0%) | 0 (0.0%) | 0 (0.0%) | 5 (13.2%) |
| Sixth year | 0 (0.0%) | 0 (0.0%) | 0 (0.0%) | 0 (0.0%) | 0 (0.0%) | 0 (0.0%) | 0 (0.0%) |
|  |  |  |  |  |  |  |  |
| **Root canal retreatments** |  |  |  |  |  |  |  |
| Non | 0 (0.0%) | 0 (0.0%) | 0 (0.0%) | 0 (0.0%) | 0 (0.0%) | 0 (0.0%) | 0 (0.0%) |
| First year | 1 (8.3%) | 0 (0.0%) | 0 (0.0%) | 0 (0.0%) | 0 (0.0%) | 1 (33.3%) | 2 (5.3%) |
| Second year | 2 (16.7%) | 1 (14.3%) | 1 (14.3%) | 2 (33.3%) | 2 (66.7%) | 0 (0.0%) | 8 (21.1%) |
| Third year | 3 (25.0%) | 1 (14.3%) | 2 (28.6%) | 1 (16.7%) | 0 (0.0%) | 1 (33.3%) | 8 (21.1%) |
| Fourth year | 4 (33.3%) | 4 (57.1%) | 5 (71.4%) | 3 (50.0%) | 1 (33.3%) | 1 (33.3%) | 18 (47.4%) |
| Fifth year | 7 (58.3%) | 4 (57.1%) | 3 (42.9%) | 0 (0.0%) | 0 (0.0%) | 0 (0.0%) | 14 (36.8%) |
| Sixth year | 1 (8.3%) | 0 (0.0%) | 0 (0.0%) | 0 (0.0%) | 0 (0.0%) | 0 (0.0%) | 1 (2.6%) |
|  |  |  |  |  |  |  |  |
| **Surgical endodontic** |  |  |  |  |  |  |  |
| Non | 0 (0.0%) | 0 (0.0%) | 0 (0.0%) | 1 (16.7%) | 1 (33.3%) | 0 (0.0%) | 2 (5.3%) |
| First year | 0 (0.0%) | 0 (0.0%) | 0 (0.0%) | 0 (0.0%) | 0 (0.0%) | 1 (33.3%) | 1 (2.6%) |
| Second year | 2 (16.7%) | 1 (14.3%) | 1 (14.3%) | 1 (16.7%) | 1 (33.3%) | 0 (0.0%) | 6 (15.8%) |
| Third year | 3 (25.0%) | 0 (0.0%) | 2 (28.6%) | 2 (33.3%) | 0 (0.0%) | 0 (0.0%) | 7 (18.4%) |
| Fourth year | 2 (16.7%) | 3 (42.9%) | 2 (28.6%) | 2 (33.3%) | 1 (33.3%) | 2 (66.7%) | 12 (31.6%) |
| Fifth year | 8 (66.7%) | 5 (71.4%) | 3 (42.9%) | 0 (0.0%) | 0 (0.0%) | 0 (0.0%) | 16 (42.1%) |
| Sixth year | 1 (8.3%) | 0 (0.0%) | 0 (0.0%) | 0 (0.0%) | 0 (0.0%) | 0 (0.0%) | 1 (2.6%) |
|  |  |  |  |  |  |  |  |
| **Endodontic regeneration** |  |  |  |  |  |  |  |
| Non | 1 (8.3%) | 0 (0.0%) | 1 (14.3%) | 0 (0.0%) | 0 (0.0%) | 0 (0.0%) | 2 (5.3%) |
| First year | 0 (0.0%) | 0 (0.0%) | 0 (0.0%) | 0 (0.0%) | 0 (0.0%) | 1 (33.3%) | 1 (2.6%) |
| Second year | 2 (16.7%) | 1 (14.3%) | 0 (0.0%) | 0 (0.0%) | 1 (33.3%) | 0 (0.0%) | 4 (10.5%) |
| Third year | 3 (25.0%) | 0 (0.0%) | 2 (28.6%) | 3 (50.0%) | 0 (0.0%) | 0 (0.0%) | 8 (21.1%) |
| Fourth year | 1 (8.3%) | 4 (57.1%) | 2 (28.6%) | 3 (50.0%) | 2 (66.7%) | 2 (66.7%) | 14 (36.8%) |
| Fifth year | 6 (50.0%) | 5 (71.4%) | 2 (28.6%) | 0 (0.0%) | 0 (0.0%) | 0 (0.0%) | 13 (34.2%) |
| Sixth year | 3 (25.0%) | 0 (0.0%) | 0 (0.0%) | 0 (0.0%) | 0 (0.0%) | 0 (0.0%) | 3 (7.9%) |
|  |  |  |  |  |  |  |  |
| **Restoration of endodontic ally treated teeth** |  |  |  |  |  |  |  |
| Non | 0 (0.0%) | 0 (0.0%) | 0 (0.0%) | 0 (0.0%) | 0 (0.0%) | 0 (0.0%) | 0 (0.0%) |
| First year | 1 (8.3%) | 0 (0.0%) | 0 (0.0%) | 0 (0.0%) | 0 (0.0%) | 1 (33.3%) | 2 (5.3%) |
| Second year | 2 (16.7%) | 1 (14.3%) | 1 (14.3%) | 3 (50.0%) | 3 (100.0%) | 0 (0.0%) | 10 (26.3%) |
| Third year | 1 (8.3%) | 0 (0.0%) | 6 (85.7%) | 3 (50.0%) | 0 (0.0%) | 0 (0.0%) | 10 (26.3%) |
| Fourth year | 6 (50.0%) | 4 (57.1%) | 3 (42.9%) | 2 (33.3%) | 1 (33.3%) | 2 (66.7%) | 18 (47.4%) |
| Fifth year | 6 (50.0%) | 4 (57.1%) | 2 (28.6%) | 0 (0.0%) | 0 (0.0%) | 0 (0.0%) | 12 (31.6%) |
| Sixth year | 0 (0.0%) | 0 (0.0%) | 0 (0.0%) | 0 (0.0%) | 0 (0.0%) | 0 (0.0%) | 0 (0.0%) |
|  |  |  |  |  |  |  |  |
|  |  |  |  |  |  |  |  |
| **Bleaching of endodontically treated teeth** |  |  |  |  |  |  |  |
| Non | 0 (0.0%) | 0 (0.0%) | 0 (0.0%) | 0 (0.0%) | 0 (0.0%) | 0 (0.0%) | 0 (0.0%) |
| First year | 0 (0.0%) | 0 (0.0%) | 0 (0.0%) | 0 (0.0%) | 0 (0.0%) | 1 (33.3%) | 1 (2.6%) |
| Second year | 2 (16.7%) | 1 (14.3%) | 0 (0.0%) | 2 (33.3%) | 1 (33.3%) | 0 (0.0%) | 6 (15.8%) |
| Third year | 2 (16.7%) | 0 (0.0%) | 2 (28.6%) | 3 (50.0%) | 0 (0.0%) | 0 (0.0%) | 7 (18.4%) |
| Fourth year | 5 (41.7%) | 4 (57.1%) | 5 (71.4%) | 3 (50.0%) | 2 (66.7%) | 2 (66.7%) | 21 (55.3%) |
| Fifth year | 4 (33.3%) | 4 (57.1%) | 1 (14.3%) | 0 (0.0%) | 0 (0.0%) | 0 (0.0%) | 9 (23.7%) |
| Sixth year | 1 (8.3%) | 0 (0.0%) | 0 (0.0%) | 0 (0.0%) | 0 (0.0%) | 0 (0.0%) | 1 (2.6%) |
|  |  |  |  |  |  |  |  |
| **Dental trauma** |  |  |  |  |  |  |  |
| Non | 1 (8.3%) | 0 (0.0%) | 0 (0.0%) | 0 (0.0%) | 0 (0.0%) | 0 (0.0%) | 1 (2.6%) |
| First year | 1 (8.3%) | 1 (14.3%) | 0 (0.0%) | 0 (0.0%) | 0 (0.0%) | 1 (33.3%) | 3 (7.9%) |
| Second year | 3 (25.0%) | 0 (0.0%) | 1 (14.3%) | 2 (33.3%) | 2 (66.7%) | 0 (0.0%) | 8 (21.1%) |
| Third year | 2 (16.7%) | 0 (0.0%) | 4 (57.1%) | 4 (66.7%) | 1 (33.3%) | 0 (0.0%) | 11 (28.9%) |
| Fourth year | 5 (41.7%) | 4 (57.1%) | 4 (57.1%) | 2 (33.3%) | 1 (33.3%) | 2 (66.7%) | 18 (47.4%) |
| Fifth year | 6 (50.0%) | 5 (71.4%) | 2 (28.6%) | 0 (0.0%) | 0 (0.0%) | 0 (0.0%) | 13 (34.2%) |
| Sixth year | 0 (0.0%) | 0 (0.0%) | 0 (0.0%) | 0 (0.0%) | 0 (0.0%) | 0 (0.0%) | 0 (0.0%) |
|  |  |  |  |  |  |  |  |
| **Endodontic emergencies** |  |  |  |  |  |  |  |
| Non | 0 (0.0%) | 0 (0.0%) | 0 (0.0%) | 0 (0.0%) | 0 (0.0%) | 1 (33.3%) | 1 (2.6%) |
| First year | 1 (8.3%) | 0 (0.0%) | 1 (14.3%) | 0 (0.0%) | 0 (0.0%) | 0 (0.0%) | 2 (5.3%) |
| Second year | 2 (16.7%) | 1 (14.3%) | 0 (0.0%) | 2 (33.3%) | 3 (100.0%) | 0 (0.0%) | 8 (21.1%) |
| Third year | 4 (33.3%) | 0 (0.0%) | 4 (57.1%) | 5 (83.3%) | 1 (33.3%) | 1 (33.3%) | 15 (39.5%) |
| Fourth year | 4 (33.3%) | 5 (71.4%) | 4 (57.1%) | 0 (0.0%) | 0 (0.0%) | 1 (33.3%) | 14 (36.8%) |
| Fifth year | 7 (58.3%) | 4 (57.1%) | 2 (28.6%) | 0 (0.0%) | 0 (0.0%) | 0 (0.0%) | 13 (34.2%) |
| Sixth year | 0 (0.0%) | 0 (0.0%) | 0 (0.0%) | 0 (0.0%) | 0 (0.0%) | 1 (33.3%) | 1 (2.6%) |
|  |  |  |  |  |  |  |  |
| **Periodontic problem** |  |  |  |  |  |  |  |
| Non | 0 (0.0%) | 0 (0.0%) | 0 (0.0%) | 1 (16.7%) | 1 (33.3%) | 0 (0.0%) | 2 (5.3%) |
| First year | 1 (8.3%) | 0 (0.0%) | 0 (0.0%) | 0 (0.0%) | 0 (0.0%) | 1 (33.3%) | 2 (5.3%) |
| Second year | 3 (25.0%) | 1 (14.3%) | 2 (28.6%) | 2 (33.3%) | 1 (33.3%) | 0 (0.0%) | 9 (23.7%) |
| Third year | 2 (16.7%) | 0 (0.0%) | 2 (28.6%) | 3 (50.0%) | 1 (33.3%) | 0 (0.0%) | 8 (21.1%) |
| Fourth year | 3 (25.0%) | 3 (42.9%) | 3 (42.9%) | 1 (16.7%) | 0 (0.0%) | 1 (33.3%) | 11 (28.9%) |
| Fifth year | 8 (66.7%) | 5 (71.4%) | 3 (42.9%) | 0 (0.0%) | 0 (0.0%) | 1 (33.3%) | 17 (44.7%) |
| Sixth year | 0 (0.0%) | 0 (0.0%) | 0 (0.0%) | 0 (0.0%) | 0 (0.0%) | 0 (0.0%) | 0 (0.0%) |
|  |  |  |  |  |  |  |  |
|  |  |  |  |  |  |  |  |
| **Orthodontic problem** |  |  |  |  |  |  |  |
| Non | 2 (16.7%) | 1 (14.3%) | 0 (0.0%) | 2 (33.3%) | 2 (66.7%) | 1 (33.3%) | 8 (21.1%) |
| First year | 1 (8.3%) | 0 (0.0%) | 0 (0.0%) | 0 (0.0%) | 0 (0.0%) | 1 (33.3%) | 2 (5.3%) |
| Second year | 1 (8.3%) | 1 (14.3%) | 0 (0.0%) | 1 (16.7%) | 1 (33.3%) | 0 (0.0%) | 4 (10.5%) |
| Third year | 2 (16.7%) | 0 (0.0%) | 2 (28.6%) | 1 (16.7%) | 0 (0.0%) | 0 (0.0%) | 5 (13.2%) |
| Fourth year | 2 (16.7%) | 3 (42.9%) | 3 (42.9%) | 2 (33.3%) | 0 (0.0%) | 0 (0.0%) | 10 (26.3%) |
| Fifth year | 3 (25.0%) | 4 (57.1%) | 4 (57.1%) | 0 (0.0%) | 0 (0.0%) | 1 (33.3%) | 12 (31.6%) |
| Sixth year | 3 (25.0%) | 0 (0.0%) | 0 (0.0%) | 0 (0.0%) | 0 (0.0%) | 0 (0.0%) | 3 (7.9%) |

**Table S4** **Minimum number of canals that students perform RCT and RRCT (n, %)**

|  | **Asia (n = 12)** | | **Africa (n = 7)** | | **Europe (n = 7)** | | **Australia (n = 6)** | | **North America (n = 3)** | | **South America (n = 3)** | | **Total (n = 38)** | |
| --- | --- | --- | --- | --- | --- | --- | --- | --- | --- | --- | --- | --- | --- | --- |
|  | **PT** | **CT** | **PT** | **CT** | **PT** | **CT** | **PT** | **CT** | **PT** | **CT** | **PT** | **CT** | **PT** | **CT** |
| **The minimum number of canals that students perform RCT** | | | | | | | | | | | | | | |
| **incisors** |  |  |  |  |  |  |  |  |  |  |  |  |  |  |
| No minimum | 0 (0.0%) | 1 (8.3%) | 0 (0.0%) | 0 (0.0%) | 0 (0.0%) | 1 (14.3%) | 1 (16.7%) | 4 (66.7%) | 1 (33.3%) | 1 (33.3%) | 0 (0.0%) | 1 (33.3%) | 2 (5%) | 8 (21%) |
| 0 | 0 (0.0%) | 0 (0.0%) | 0 (0.0%) | 0 (0.0%) | 0 (0.0%) | 0 (0.0%) | 0 (0.0%) | 0 (0.0%) | 0 (0.0%) | 0 (0.0%) | 1 (33.3%) | 0 (0.0%) | 1 (3%) | 0 (0.0%) |
| ≤2 | 9 (75%) | 8 (66.7%) | 3 (43%) | 4 (57%) | 5 (71%) | 4 (57%) | 3 (50%) | 2 (33%) | 1 (33.3%) | 2 (66.7%) | 1 (33.3%) | 1 (33%) | 22 (58%) | 21 (55%) |
| >2 | 3 (25%) | 3 (25%) | 4 (57%) | 3 (43%) | 2 (29%) | 2 (28%) | 2 (33.3%) | 0 (0.0%) | 1 (33.3%) | 0 (0.0%) | 1 (33.3%) | 1 (33%) | 13 (34) | 9 (24%) |
| **Canine** |  |  |  |  |  |  |  |  |  |  |  |  |  |  |
| No minimum | 1 (8.3%) | 2 (16.7%) | 2 (28.6%) | 2 (28.6%) | 0 (0.0%) | 2 (28.6%) | 1 (16.7%) | 5 (83.3%) | 1 (33.3%) | 1 (33.3%) | 1 (33.3%) | 0 (0.0%) | 6 (15.8%) | 12 (31.6%) |
| 0 | 1 (8.3%) | 1 (8.3%) | 0 (0.0%) | 0 (0.0%) | 0 (0.0%) | 0 (0.0%) | 4 (66.7%) | 0 (0.0%) | 0 (0.0%) | 0 (0.0%) | 0 (0.0%) | 1 (33.3%) | 5 (13.2%) | 2 (5.3%) |
| ≤2 | 8 (66.6%) | 7 (58.3%) | 5 (71.4%) | 4 (57%) | 5 (71.4) | 3 (43%) | 1 (16.6%) | 1 (16.7%) | 2 (66.6) | 2 (66.7%) | 1 (33.3%) | 1 (33.3%) | 22 (58%) | 19 (47%) |
| >2 | 2 (16.6%) | 2 (16.6%) | 0 (0.0%) | 1 (14.3%) | 2 (28.5%) | 2 (28.6%) | 0 (0.0%) | 0 (0.0%) | 0 (0.0%) | 0 (0.0%) | 1 (33.3%) | 1 (33.3%) | 5 (13%) | 6 (16%) |
| **Premolars** |  |  |  |  |  |  |  |  |  |  |  |  |  |  |
| No minimum | 0 (0.0%) | 0 (0.0%) | 1 (14.3%) | 1 (0.0%) | 0 (0.0%) | 2 (0.0%) | 2 (33.3%) | 5 (83.3%) | 1 (33.3%) | 0 (0.0%) | 1 (33.3%) | 0 (0.0%) | 5 (13.2%) | 8 (21%) |
| 0 | 0 (0.0%) | 0 (0.0%) | 0 (0.0%) | 0 (0.0%) | 0 (0.0%) | 0 (0.0%) | 0 (0.0%) | 0 (0.0%) | 0 (0.0%) | 0 (0.0%) | 0 (0.0%) | 1 (33.3%) | 0 (0.0%) | 1 (3%) |
| ≤2 | 11 (91.7%) | 9 (75%) | 4 (57%) | 4 (57.2%) | 5 (71.4%) | 4 (57.2%) | 3 (50%) | 1 (16.7%) | 2 (66.6) | 2 (66.6%) | 1 (33.3%) | 1 (33.3%) | 26 (68.4%) | 21 (55%) |
| >2 | 1 (8.3%) | 3 (24.9%) | 2 (28.5%) | 2 (28.6%) | 2 (28.5%) | 2 (28.6%) | 0 (0.0%) | 0 (0.0%) | 0 (0.0%) | 0 (0.0%) | 1 (33.3%) | 1 (33.3%) | 7 (18.4%) | 8 (21%) |
| Molars |  |  |  |  |  |  |  |  |  |  |  |  |  |  |
| **No minimum** | 0 (0.0%) | 0 (0.0%) | 0 (0.0%) | 0 (0.0%) | 0 (0.0%) | 0 (0.0%) | 1 (16.7%) | 4 (66.7%) | 1 (33.3%) | 0 (0.0%) | 0 (0.0%) | 0 (0.0%) | 2 (5.3%) | 4 (10.5%) |
| 0 | 0 (0.0%) | 0 (0.0%) | 1 (14.3%) | 1 (14.3%) | 0 (0.0%) | 0 (0.0%) | 0 (0.0%) | 1 (16.7%) | 0 (0.0%) | 0 (0.0%) | 2 (66.7%) | 2 (66.7%) | 3 (8%) | 4 (10.5%) |
| ≤2 | 11 (99.6%) | 9 (75%) | 6 (85.7%) | 3 (42.9%) | 5 (71.4%) | 3 (42.9) | 1(16.6%) | 1 (16.7%) | 1 (33.3%) | 3 (100.0%) | 1 (33.3%) | 0 (0.0%) | 25 (65.7%) | 19 (50%) |
| >2 | 1 (8.3%) | 3 (24.9%) | 0 (0.0%) | 3 (42.9%) | 2 (28.5%) | 4 (57.2%) | 4 (66.6%) | 0 (0.0%) | 1 (33.3%) | 0 (0.0%) | 0 (0.0%) | 1 (33.3%) | 6 (21%) | 11 (29%) |
|  |  |  |  |  |  |  |  |  |  |  |  |  |  |  |
| **The minimum number of canals that students perform RRCT** | | | | | | | | | | | | | | |
| **Incisors** |  |  |  |  |  |  |  |  |  |  |  |  |  |  |
| No minimum | 0 (0.0%) | 2 (16.7%) | 1 (14.3%) | 3 (42.9%) | 0 (0.0%) | 1 (14.3%) | 0 (0.0%) | 2 (33.3%) | 0 (0.0%) | 0 (0.0%) | 0 (0.0%) | 0 (0.0%) | 0 (0.0%) | 8 (21.1%) |
| 0 | 10 (83.3%) | 7 (58.3%) | 5 (71.4%) | 2 (28.6%) | 7 (100.0%) | 4 (57.1%) | 5 (83.3%) | 4 (66.7%) | 3 (100.0%) | 3 (100.0%) | 2 (66.7%) | 1 (33.3%) | 32 (84.2%) | 21 (55.3%) |
| 1 | 2 (16.7%) | 2 (16.7%) | 1 (14.3%) | 2 (28.6%) | 0 (0.0%) | 1 (14.3%) | 1 (16.7%) | 0 (0.0%) | 0 (0.0%) | 0 (0.0%) | 1 (33.3%) | 2 (66.7%) | 5 (13.2%) | 7 (18.4%) |
| 2 | 0 (0.0%) | 1 (8.3%) | 1 (14.3%) | 0 (0.0%) | 0 (0.0%) | 1 (14.3%) | 0 (0.0%) | 0 (0.0%) | 0 (0.0%) | 0 (0.0%) | 0 (0.0%) | 0 (0.0%) | 1 (2.6%) | 2 (5.3%) |
| No minimum |  |  |  |  |  |  |  |  |  |  |  |  |  |  |
| **Canines** | 0 (0.0%) | 2 (16.7%) | 1 (14.3%) | 3 (42.9%) | 0 (0.0%) | 1 (14.3%) | 0 (0.0%) | 2 (33.3%) | 0 (0.0%) | 0 (0.0%) | 0 (0.0%) | 0 (0.0%) | 0 (0.0%) | 8 (21.1%) |
| 0 | 10 (83.3%) | 7 (58.3%) | 6 (85.7%) | 3 (42.9%) | 7 (100.0%) | 4 (57.1%) | 6 (100.0%) | 4 (66.7%) | 3 (100.0%) | 3 (100.0%) | 1 (33.3%) | 1 (33.3%) | 33 (86.8%) | 22 (57.9%) |
| 1 | 2 (16.7%) | 2 (16.7%) | 0 (0.0%) | 1 (14.3%) | 0 (0.0%) | 1 (14.3%) | 0 (0.0%) | 0 (0.0%) | 0 (0.0%) | 0 (0.0%) | 2 (66.7%) | 2 (66.7%) | 4 (10.5%) | 6 (15.8%) |
| 2 | 0 (0.0%) | 1 (8.3%) | 1 (14.3%) | 0 (0.0%) | 0 (0.0%) | 1 (14.3%) | 0 (0.0%) | 0 (0.0%) | 0 (0.0%) | 0 (0.0%) | 0 (0.0%) | 0 (0.0%) | 1 (2.6%) | 2 (5.3%) |
| **Premolars** |  |  |  |  |  |  |  |  |  |  |  |  |  |  |
| No minimum | 0 (0.0%) | 2 (16.7%) | 1 (14.3%) | 3 (42.9%) | 0 (0.0%) | 1 (14.3%) | 0 (0.0%) | 2 (33.3%) | 0 (0.0%) | 0 (0.0%) | 0 (0.0%) | 0 (0.0%) | 0 (0.0%) | 8 (21.1%) |
| 0 | 10 (83.3%) | 7 (58.3%) | 6 (85.7%) | 3 (42.9%) | 7 (100.0%) | 4 (57.1%) | 6 (100.0%) | 4 (66.7%) | 3 (100.0%) | 3 (100.0%) | 1 (33.3%) | 1 (33.3%) | 33 (86.8%) | 22 (57.9%) |
| 1 | 2 (16.7%) | 2 (16.7%) | 0 (0.0%) | 1 (14.3%) | 0 (0.0%) | 1 (14.3%) | 0 (0.0%) | 0 (0.0%) | 0 (0.0%) | 0 (0.0%) | 2 (66.7%) | 2 (66.7%) | 4 (10.5%) | 6 (15.8%) |
| 2 | 0 (0.0%) | 1 (8.3%) | 1 (14.3%) | 0 (0.0%) | 0 (0.0%) | 1 (14.3%) | 0 (0.0%) | 0 (0.0%) | 0 (0.0%) | 0 (0.0%) | 0 (0.0%) | 0 (0.0%) | 1 (2.6%) | 2 (5.3%) |
| **Molars** |  |  |  |  |  |  |  |  |  |  |  |  |  |  |
| No minimum | 0 (0.0%) | 2 (16.7%) | 1 (14.3%) | 3 (42.9%) | 0 (0.0%) | 1 (14.3%) | 0 (0.0%) | 2 (33.3%) | 0 (0.0%) | 0 (0.0%) | 0 (0.0%) | 0 (0.0%) | 0 (0.0%) | 8 (21.1%) |
| 0 | 11 (91.7%) | 9 (75.0%) | 6 (85.7%) | 3 (42.9%) | 6 (85.7%) | 4 (57.1%) | 6 (100.0%) | 4 (66.7%) | 3 (100.0%) | 3 (100.0%) | 3 (100.0%) | 2 (66.7%) | 35 (92.1%) | 25 (65.8%) |
| 1 | 1 (8.3%) | 0 (0.0%) | 1 (14.3%) | 1 (14.3%) | 1 (14.3%) | 1 (14.3%) | 0 (0.0%) | 0 (0.0%) | 0 (0.0%) | 0 (0.0%) | 0 (0.0%) | 1 (33.3%) | 3 (7.9%) | 3 (7.9%) |
| 2 | 0 (0.0%) | 1 (8.3%) | 1 (14.3%) | 0 (0.0%) | 0 (0.0%) | 1 (14.3%) | 0 (0.0%) | 0 (0.0%) | 0 (0.0%) | 0 (0.0%) | 0 (0.0%) | 0 (0.0%) | 0 (0.0%) | 2 (5.3%) |

**RRCT: Retreatment**

**Table S5: Techniques details (n, %)**

|  | **Asia (n = 12)** | | **Africa (n = 7)** | | **Europe (n = 7)** | | **Australia (n = 6)** | | **North America (n = 3)** | | **South America (n = 3)** | | **Total (n = 38)** | |
| --- | --- | --- | --- | --- | --- | --- | --- | --- | --- | --- | --- | --- | --- | --- |
|  | **PT** | **CT** | **PT** | **CT** | **PT** | **CT** | **PT** | **CT** | **PT** | **CT** | **PT** | **CT** | **PT** | **CT** |
| **Magnifying system** | | | | | | | | | | | | | | |
| Magnifying systems are not used | 8 (66.7%) | 8 (66.6%) | 4 (57.1%) | 4 (57.1%) | 3 (42.9%) | 2 (28.6%) | 0 (0.0%) | 0 (0.0%) | 0 (0.0%) | 0 (0.0%) | 1 (33.3%) | 1 (33.3%) | 16 (42.1%) | 14 (36.8%) |
| Dental loupes | 4 (33.3%) | 4 (33.3%) | 3 (42.9%) | 3 (42.9%) | 4 (57.1%) | 4 (57.1%) | 4 (66.7%) | 5 (83.3%) | 1 (33.3%) | 1 (33.3%) | 1 (33.3%) | 1 (33.3%) | 17 (44.7%) | 18 (47.4%) |
| Microscope | 0 (0.0%) | 0 (0.0%) | 0 (0.0%) | 0 (0.0%) | 0 (0.0%) | 0 (0.0%) | 0 (0.0%) | 0 (0.0%) | 0 (0.0%) | 0 (0.0%) | 1 (33.3%) | 1 (33.3%) | 1 (2.6%) | 1 (2.6%) |
| Dental loupes and Microscope | 0 (0.0%) | 0 (0.0%) | 0 (0.0%) | 0 (0.0%) | 0 (0.0%) | 1 (14.3%) | 2 (33.3%) | 1 (16.7%) | 2 (66.7%) | 2 (66.7%) | 0 (0.0%) | 0 (0.0%) | 4 (10.5%) | 4 (10.5%) |
|  |  |  |  |  |  |  |  |  |  |  |  |  |  |  |
| **Ultrasonic instruments** | | | | | | | | | | | | | | |
| Yes | 0 (0.0%) | 2 (16.7%) | 0 (0.0%) | 1 (14.3%) | 0 (0.0%) | 2 (28.6%) | 1 (16.7%) | 2 (33.3%) | 0 (0.0%) | 1 (33.3%) | 1 (33.3%) | 1 (33.3%) | 2 (5.3%) | 9 (23.7%) |
| No | 12 (100.0%) | 10 (83.3%) | 7 (100.0%) | 6 (85.7%) | 7 (100.0%) | 5 (71.4%) | 5 (83.3%) | 4 (66.7%) | 3 (100.0%) | 2 (66.7%) | 2 (66.7%) | 2 (66.7%) | 36 (94.7%) | 29 (76.3%) |
|  |  |  |  |  |  |  |  |  |  |  |  |  |  |  |
| **Working length determination** | | | | | | | | | | | | | | |
| Radiographs only | 1 (8.3%) | 1 (8.3%) | 1 (14.3%) | 2 (28.6%) | 2 (28.6%) | 0 (0.0%) | 0 (0.0%) | 0 (0.0%) | 0 (0.0%) | 0 (0.0%) | 0 (0.0%) | 2 (66.7%) | 4 (10.5%) | 5 (13.2%) |
| Electronic apex locator only | 9 (75.0%) | 1 (8.3%) | 4 (57.1%) | 0 (0.0%) | 2 (28.6%) | 0 (0.0%) | 4 (66.7%) | 1 (16.7%) | 2 (66.7%) | 0 (0.0%) | 2 (66.7%) | 0 (0.0%) | 23 (60.5%) | 2 (5.3%) |
| Both radiographs and electronic apex locator | 2 (16.7%) | 10 (83.3%) | 2 (28.6%) | 5 (71.4%) | 3 (42.9%) | 7 (100.0%) | 2 (33.3%) | 5 (83.3%) | 1 (33.3%) | 3 (100.0%) | 1 (33.3%) | 1 (33.3%) | 11 (28.9%) | 31 (81.6%) |
|  |  |  |  |  |  |  |  |  |  |  |  |  |  |  |
| **Instruments used for root canal shaping** | | | | | | | | | | | | | | |
| Manual stainless instruments | 12 (100.0%) | 12 (100.0%) | 7 (100.0%) | 7 (100.0%) | 5 (71.4%) | 5 (71.4%) | 4 (66.7%) | 5 (83.3%) | 1 (33.3%) | 1 (33.3%) | 3 (100.0%) | 3 (100.0%) | 32 (84.2%) | 33 (86.8%) |
| Manual Nickel- titanium instruments | 4 (33.3%) | 6 (50.0%) | 4 (57.1%) | 3 (42.9%) | 4 (57.1%) | 4 (57.1%) | 0 (0.0%) | 0 (0.0%) | 1 (33.3%) | 1 (33.3%) | 2 (66.7%) | 2 (66.7%) | 15 (39.5%) | 16 (42.1%) |
| Engine driven rotary NiTi ﬁles | 8 (66.7%) | 10 (83.3%) | 4 (57.1%) | 6 (85.7%) | 4 (57.1%) | 5 (71.4%) | 6 (100.0%) | 5 (83.3%) | 2 (66.7%) | 2 (66.7%) | 0 (0.0%) | 0 (0.0%) | 24 (63.2%) | 28 (73.7%) |
| Engine driven reciprocating NiTi ﬁles | 3 (25.0%) | 2 (16.7%) | 0 (0.0%) | 0 (0.0%) | 2 (28.6%) | 3 (42.9%) | 0 (0.0%) | 1 (16.7%) | 3 (100.0%) | 3 (100.0%) | 0 (0.0%) | 0 (0.0%) | 8 (21.1%) | 9 (23.7%) |
|  |  |  |  |  |  |  |  |  |  |  |  |  |  |  |
| **The main manual (hand) instrumentation technique of root canal preparation** | | | | | | | | | | | | | | |
| No manual technique used | 0 (0.0%) | 0 (0.0%) | 0 (0.0%) | 0 (0.0%) | 1 (14.3%) | 0 (0.0%) | 2 (33.3%) | 3 (50.0%) | 1 (33.3%) | 1 (33.3%) | 1 (33.3%) | 1 (33.3%) | 5 (13.2%) | 5 (13.2%) |
| Step back technique | 8 (66.7%) | 8 (66.7%) | 6 (85.7%) | 6 (85.7%) | 5 (71.4%) | 5 (71.4%) | 2 (33.3%) | 0 (0.0%) | 1 (33.3%) | 1 (33.3%) | 2 (66.7%) | 2 (66.7%) | 24 (63.2%) | 22 (57.9%) |
| Crown down technique | 3 (25.0%) | 5 (41.7%) | 4 (57.1%) | 4 (57.1%) | 0 (0.0%) | 3 (42.9%) | 1 (16.7%) | 1 (16.7%) | 1 (33.3%) | 1 (33.3%) | 1 (33.3%) | 1 (33.3%) | 10 (26.3%) | 15 (39.5%) |
| Balanced force technique | 0 (0.0%) | 0 (0.0%) | 0 (0.0%) | 0 (0.0%) | 0 (0.0%) | 0 (0.0%) | 2 (33.3%) | 2 (33.3%) | 0 (0.0%) | 0 (0.0%) | 1 (33.3%) | 0 (0.0%) | 3 (7.9%) | 2 (5.3%) |
| Standardized technique | 1 (8.3%) | 1 (8.3%) | 1 (14.3%) | 2 (28.6%) | 1 (14.3%) | 1 (14.3%) | 1 (16.7%) | 1 (16.7%) | 0 (0.0%) | 0 (0.0%) | 2 (66.7%) | 2 (66.7%) | 6 (15.8%) | 7 (18.4%) |
| Hybrid technique (step back and crown down techniques) | 1 (8.3%) | 3 (25.0%) | 2 (28.6%) | 3 (42.9%) | 1 (14.3%) | 3 (42.9%) | 0 (0.0%) | 0 (0.0%) | 0 (0.0%) | 0 (0.0%) | 1 (33.3%) | 1 (33.3%) | 5 (13.2%) | 10 (26.3%) |
| Others | 0 (0.0%) | 1 (8.3%) | 0 (0.0%) | 0 (0.0%) | 0 (0.0%) | 0 (0.0%) | 1 (16.7%) | 1 (16.7%) | 0 (0.0%) | 0 (0.0%) | 0 (0.0%) | 0 (0.0%) | 1 (2.6%) | 2 (5.3%) |
|  |  |  |  |  |  |  |  |  |  |  |  |  |  |  |
| **Materials/ instruments used for removing Root Canal Filling Material in Retreatment** | | | | | | | | | | | | | | |
| Students do not perform retreatment | 10 (83.3%) | 7 (58.3%) | 5 (71.4%) | 3 (42.9%) | 7 (100.0%) | 4 (57.1%) | 5 (83.3%) | 4 (66.7%) | 3 (100.0%) | 3 (100.0%) | 1 (33.3%) | 1 (33.3%) | 31 (81.6%) | 22 (57.9%) |
| Gates Glidden drills | 2 (16.7%) | 4 (33.3%) | 2 (28.6%) | 3 (42.9%) | 0 (0.0%) | 0 (0.0%) | 1 (16.7%) | 1 (16.7%) | 0 (0.0%) | 0 (0.0%) | 1 (33.3%) | 2 (66.7%) | 6 (15.8%) | 10 (26.3%) |
| Peeso Reamers | 0 (0.0%) | 2 (16.7%) | 0 (0.0%) | 1 (14.3%) | 0 (0.0%) | 0 (0.0%) | 0 (0.0%) | 0 (0.0%) | 0 (0.0%) | 0 (0.0%) | 0 (0.0%) | 0 (0.0%) | 0 (0.0%) | 3 (7.9%) |
| Nickel-titanium rotary files | 1 (8.3%) | 4 (33.3%) | 1 (14.3%) | 4 (57.1%) | 0 (0.0%) | 3 (42.9%) | 1 (16.7%) | 2 (33.3%) | 0 (0.0%) | 0 (0.0%) | 1 (33.3%) | 0 (0.0%) | 4 (10.5%) | 13 (34.2%) |
| Hedstrom file (H-F)) | 2 (16.7%) | 4 (33.3%) | 2 (28.6%) | 4 (57.1%) | 0 (0.0%) | 1 (14.3%) | 0 (0.0%) | 0 (0.0%) | 0 (0.0%) | 0 (0.0%) | 0 (0.0%) | 1 (33.3%) | 4 (10.5%) | 10 (26.3%) |
| Xylene | 0 (0.0%) | 1 (8.3%) | 0 (0.0%) | 0 (0.0%) | 0 (0.0%) | 1 (14.3%) | 0 (0.0%) | 1 (16.7%) | 0 (0.0%) | 0 (0.0%) | 1 (33.3%) | 0 (0.0%) | 1 (2.6%) | 3 (7.9%) |
| Chloroform | 1 (8.3%) | 3 (25.0%) | 1 (14.3%) | 3 (42.9%) | 0 (0.0%) | 0 (0.0%) | 1 (16.7%) | 1 (16.7%) | 0 (0.0%) | 0 (0.0%) | 0 (0.0%) | 0 (0.0%) | 3 (7.9%) | 7 (18.4%) |
| Eucalyptol | 0 (0.0%) | 0 (0.0%) | 1 (14.3%) | 1 (14.3%) | 0 (0.0%) | 2 (28.6%) | 0 (0.0%) | 0 (0.0%) | 0 (0.0%) | 0 (0.0%) | 1 (33.3%) | 1 (33.3%) | 2 (5.3%) | 4 (10.5%) |
| Halothane | 0 (0.0%) | 0 (0.0%) | 0 (0.0%) | 0 (0.0%) | 0 (0.0%) | 0 (0.0%) | 0 (0.0%) | 0 (0.0%) | 0 (0.0%) | 0 (0.0%) | 0 (0.0%) | 0 (0.0%) | 0 (0.0%) | 0 (0.0%) |
| Endosolv (Ethyl acetate) | 0 (0.0%) | 0 (0.0%) | 0 (0.0%) | 0 (0.0%) | 0 (0.0%) | 1 (14.3%) | 0 (0.0%) | 0 (0.0%) | 0 (0.0%) | 0 (0.0%) | 0 (0.0%) | 0 (0.0%) | 0 (0.0%) | 1 (2.6%) |
| None | 0 (0.0%) | 0 (0.0%) | 0 (0.0%) | 0 (0.0%) | 0 (0.0%) | 0 (0.0%) | 0 (0.0%) | 0 (0.0%) | 0 (0.0%) | 0 (0.0%) | 1 (33.3%) | 0 (0.0%) | 1 (2.6%) | 0 (0.0%) |
|  |  |  |  |  |  |  |  |  |  |  |  |  |  |  |
|  |  |  |  |  |  |  |  |  |  |  |  |  |  |  |
| **Irrigation solution** | | | | | | | | | | | | | | |
| NaOCl | 9 (75.0%) | 12 (100.0%) | 7 (100.0%) | 7 (100.0%) | 5 (71.4%) | 7 (100.0%) | 1 (16.7%) | 6 (100.0%) | 2 (66.7%) | 3 (100.0%) | 2 (66.7%) | 2 (66.7%) | 26 (68.4%) | 37 (97.4%) |
| CHX | 3 (25.0%) | 4 (33.3%) | 1 (14.3%) | 3 (42.9%) | 1 (14.3%) | 4 (57.1%) | 0 (0.0%) | 0 (0.0%) | 0 (0.0%) | 0 (0.0%) | 1 (33.3%) | 2 (66.7%) | 6 (15.8%) | 13 (34.2%) |
| (EDTA) | 4 (33.3%) | 7 (58.3%) | 4 (57.1%) | 6 (85.7%) | 1 (14.3%) | 5 (71.4%) | 2 (33.3%) | 4 (66.7%) | 1 (33.3%) | 2 (66.7%) | 1 (33.3%) | 1 (33.3%) | 13 (34.2%) | 25 (65.8%) |
| Citric acid | 0 (0.0%) | 0 (0.0%) | 0 (0.0%) | 0 (0.0%) | 1 (14.3%) | 1 (14.3%) | 0 (0.0%) | 0 (0.0%) | 0 (0.0%) | 0 (0.0%) | 0 (0.0%) | 0 (0.0%) | 1 (2.6%) | 1 (2.6%) |
| Ethanol | 0 (0.0%) | 0 (0.0%) | 0 (0.0%) | 0 (0.0%) | 0 (0.0%) | 0 (0.0%) | 0 (0.0%) | 0 (0.0%) | 0 (0.0%) | 0 (0.0%) | 0 (0.0%) | 0 (0.0%) | 0 (0.0%) | 0 (0.0%) |
| Local anesthetic solution | 0 (0.0%) | 0 (0.0%) | 0 (0.0%) | 0 (0.0%) | 0 (0.0%) | 0 (0.0%) | 0 (0.0%) | 0 (0.0%) | 0 (0.0%) | 0 (0.0%) | 0 (0.0%) | 0 (0.0%) | 0 (0.0%) | 0 (0.0%) |
| Normal saline | 7 (58.3%) | 7 (58.3%) | 2 (28.6%) | 1 (14.3%) | 3 (42.9%) | 1 (14.3%) | 0 (0.0%) | 0 (0.0%) | 0 (0.0%) | 0 (0.0%) | 1 (33.3%) | 1 (33.3%) | 13 (34.2%) | 1 (2.6%) |
| Water | 3 (25.0%) | 0 (0.0%) | 0 (0.0%) | 0 (0.0%) | 3 (42.9%) | 0 (0.0%) | 5 (83.3%) | 0 (0.0%) | 1 (33.3%) | 0 (0.0%) | 1 (33.3%) | 1 (33.3%) | 13 (34.2%) | 10 (26.3%) |
|  |  |  |  |  |  |  |  |  |  |  |  |  |  |  |
| **Irrigation technique** | | | | | | | | | | | | | | |
| Passive needle irrigation | 11 (91.7%) | 10 (83.3%) | 6 (85.7%) | 5 (71.4%) | 7 (100.0%) | 7 (100.0%) | 5 (83.3%) | 5 (83.3%) | 3 (100.0%) | 3 (100.0%) | 2 (66.7%) | 2 (66.7%) | 34 (89.5%) | 32 (84.2%) |
| Manual agitation using gutta percha | 3 (25.0%) | 5 (41.7%) | 3 (42.9%) | 3 (42.9%) | 2 (28.6%) | 3 (42.9%) | 2 (33.3%) | 2 (33.3%) | 0 (0.0%) | 0 (0.0%) | 0 (0.0%) | 0 (0.0%) | 10 (26.3%) | 13 (34.2%) |
| Dynamic irrigation using sonic ultrasound energy | 0 (0.0%) | 2 (16.7%) | 0 (0.0%) | 1 (14.3%) | 1 (14.3%) | 2 (28.6%) | 0 (0.0%) | 0 (0.0%) | 1 (33.3%) | 1 (33.3%) | 1 (33.3%) | 0 (0.0%) | 3 (7.9%) | 6 (15.8%) |
| Dynamic irrigation using ultrasonic energy | 0 (0.0%) | 0 (0.0%) | 0 (0.0%) | 0 (0.0%) | 0 (0.0%) | 0 (0.0%) | 0 (0.0%) | 0 (0.0%) | 0 (0.0%) | 0 (0.0%) | 0 (0.0%) | 1 (33.3%) | 0 (0.0%) | 1 (2.6%) |
|  |  |  |  |  |  |  |  |  |  |  |  |  |  |  |
| **The main method for root canal obturation** | | | | | | | | | | | | | | |
| Single cone technique | 7 (58.3%) | 7 (58.3%) | 2 (28.6%) | 4 (57.1%) | 0 (0.0%) | 2 (28.6%) | 3 (50.0%) | 3 (50.0%) | 1 (33.3%) | 1 (33.3%) | 2 (66.7%) | 2 (66.7%) | 15 (39.5%) | 19 (50.0%) |
| Cold lateral compaction | 12 (100.0%) | 12 (100.0%) | 6 (85.7%) | 5 (71.4%) | 7 (100.0%) | 7 (100.0%) | 5 (83.3%) | 4 (66.7%) | 1 (33.3%) | 1 (33.3%) | 2 (66.7%) | 2 (66.7%) | 33 (86.8%) | 31 (81.6%) |
| Warm vertical compaction  (Schilder technique) | 0 (0.0%) | 0 (0.0%) | 0 (0.0%) | 1 (14.3%) | 0 (0.0%) | 1 (14.3%) | 1 (16.7%) | 1 (16.7%) | 0 (0.0%) | 0 (0.0%) | 0 (0.0%) | 0 (0.0%) | 1 (2.6%) | 3 (7.9%) |
| Warm vertical compaction using continuous technique (Buchanan) | 0 (0.0%) | 0 (0.0%) | 0 (0.0%) | 1 (14.3%) | 0 (0.0%) | 1 (14.3%) | 0 (0.0%) | 1 (16.7%) | 1 (33.3%) | 1 (33.3%) | 1 (33.3%) | 1 (33.3%) | 2 (5.3%) | 5 (13.2%) |
| Thermoplastic injection technique | 0 (0.0%) | 1 (8.3%) | 0 (0.0%) | 1 (14.3%) | 0 (0.0%) | 0 (0.0%) | 0 (0.0%) | 0 (0.0%) | 1 (33.3%) | 1 (33.3%) | 0 (0.0%) | 0 (0.0%) | 1 (2.6%) | 3 (7.9%) |
| Carrier-based technique | 0 (0.0%) | 0 (0.0%) | 0 (0.0%) | 0 (0.0%) | 0 (0.0%) | 0 (0.0%) | 0 (0.0%) | 0 (0.0%) | 0 (0.0%) | 0 (0.0%) | 0 (0.0%) | 0 (0.0%) | 0 (0.0%) | 0 (0.0%) |
| Paste ﬁllers | 0 (0.0%) | 0 (0.0%) | 1 (14.3%) | 1 (14.3%) | 0 (0.0%) | 0 (0.0%) | 0 (0.0%) | 0 (0.0%) | 0 (0.0%) | 0 (0.0%) | 2 (66.7%) | 3 (100.0%) | 3 (7.9%) | 4 (10.5%) |
| Thermo-mechanic compaction | 0 (0.0%) | 0 (0.0%) | 0 (0.0%) | 1 (14.3%) | 0 (0.0%) | 0 (0.0%) | 0 (0.0%) | 0 (0.0%) | 1 (33.3%) | 1 (33.3%) | 1 (33.3%) | 0 (0.0%) | 2 (5.3%) | 2 (5.3%) |
| Other | 0 (0.0%) | 0 (0.0%) | 1 (14.3%) | 0 (0.0%) | 0 (0.0%) | 0 (0.0%) | 0 (0.0%) | 1 (16.7%) | 0 (0.0%) | 0 (0.0%) | 0 (0.0%) | 0 (0.0%) | 1 (2.6%) | 1 (2.6%) |
|  |  |  |  |  |  |  |  |  |  |  |  |  |  |  |
| **Advanced endodontic materials/technologies** | | | | | | | | | | | | | | |
| Mineral trioxide aggregate (MTA) | 3 (25.0%) | 7 (58.3%) | 1 (14.3%) | 5 (71.4%) | 1 (14.3%) | 3 (42.9%) | 1 (16.7%) | 1 (16.7%) | 0 (0.0%) | 0 (0.0%) | 1 (33.3%) | 2 (66.7%) | 7 (18.4%) | 18 (47.4%) |
| Biodentine | 2 (16.7%) | 3 (25.0%) | 1 (14.3%) | 1 (14.3%) | 1 (14.3%) | 3 (42.9%) | 0 (0.0%) | 0 (0.0%) | 0 (0.0%) | 0 (0.0%) | 1 (33.3%) | 0 (0.0%) | 5 (13.2%) | 7 (18.4%) |
| Bioceramic sealers | 3 (25.0%) | 5 (41.7%) | 0 (0.0%) | 2 (28.6%) | 1 (14.3%) | 2 (28.6%) | 0 (0.0%) | 0 (0.0%) | 0 (0.0%) | 0 (0.0%) | 0 (0.0%) | 0 (0.0%) | 4 (10.5%) | 9 (23.7%) |
| Cone beam computed tomography (CBCT) | 1 (8.3%) | 5 (41.7%) | 0 (0.0%) | 1 (14.3%) | 0 (0.0%) | 0 (0.0%) | 1 (16.7%) | 4 (66.7%) | 0 (0.0%) | 0 (0.0%) | 1 (33.3%) | 1 (33.3%) | 3 (7.9%) | 11 (28.9%) |
| Thermally treated nickel-titanium ﬁles | 4 (33.3%) | 5 (41.7%) | 1 (14.3%) | 2 (28.6%) | 0 (0.0%) | 0 (0.0%) | 2 (33.3%) | 3 (50.0%) | 1 (33.3%) | 1 (33.3%) | 0 (0.0%) | 0 (0.0%) | 8 (21.1%) | 11 (28.9%) |
| Guided endodontic access technology | 1 (8.3%) | 0 (0.0%) | 0 (0.0%) | 1 (14.3%) | 0 (0.0%) | 0 (0.0%) | 0 (0.0%) | 0 (0.0%) | 0 (0.0%) | 0 (0.0%) | 0 (0.0%) | 0 (0.0%) | 1 (2.6%) | 1 (2.6%) |
| Other (No advanced materials used during PT training) | 5 (41.7%) | 2 (16.7%) | 5 (71.4%) | 2 (28.6%) | 5 (71.4%) | 3 (42.9%) | 1 (16.7%) | 1 (16.7%) | 2 (66.7%) | 2 (66.7%) | 0 (0.0%) | 0 (0.0%) | 18 (47.4%) | 10 (26.3%) |

**Table S6** **Placement of intracanal medicament between CT appointments and type of restoration following root canal treatment (n, %)**

|  | **Asia (n = 12)** | **Africa (n = 7)** | **Europe (n = 7)** | **Australia (n = 6)** | **North America (n = 3)** | **South America (n = 3)** | **Total (n = 38)** |
| --- | --- | --- | --- | --- | --- | --- | --- |
| **Placement of intracanal medicament between CT appointments** | | | | | | | |
| Calcium hydroxide | 12 (100.0%) | 6 (85.7%) | 7 (100.0%) | 6 (100.0%) | 3 (100.0%) | 2 (66.7%) | 36 (94.7%) |
| Ledermix | 1 (8.3%) | 1 (14.3%) | 2 (28.6%) | 1 (16.7%) | 0 (0.0%) | 0 (0.0%) | 5 (13.2%) |
| None – single visit treatment whenever possible | 1 (8.3%) | 0 (0.0%) | 0 (0.0%) | 0 (0.0%) | 0 (0.0%) | 0 (0.0%) | 1 (2.6%) |
| based on different diagnosis | 1 (8.3%) | 2 (28.6%) | 0 (0.0%) | 1 (16.7%) | 0 (0.0%) | 1 (33.3%) | 5 (13.2%) |
| No medicament used | 0 (0.0%) | 0 (0.0%) | 0 (0.0%) | 0 (0.0%) | 0 (0.0%) | 1 (33.3%) | 1 (2.6%) |
| Other | 1 (8.3%) | 1 (14.3%) | 0 (0.0%) | 2 (33.3%) | 0 (0.0%) | 0 (0.0%) | 4 (10.5%) |
|  |  |  |  |  |  |  |  |
| **Type of restoration placed after completion of root canal treatment in the CT (n, %)** | | | | | | | |
| Provisional (temporary) restoration (cement ﬁlling) | 4 (33.3%) | 2 (28.6%) | 5 (71.4%) | 2 (33.3%) | 2 (66.7%) | 2 (66.7%) | 17 (44.7%) |
| Deﬁnitive restoration (adhesive ﬁlling) | 8 (66.7%) | 5 (71.4%) | 2 (28.6%) | 4 (66.7%) | 1 (33.3%) | 1 (33.3%) | 21 (55.3%) |

**CT: CT training**

**Appendix 1:**

**Please tick the following boxed to indicate you accept and agree to the following:**

My participation in the project is entirely voluntary  agree

I am free to withdraw from the project at any time without any disadvantage agree

All data will be retained in a secure storage in an anonymized format agree

No risks to participants have been identified agree

1. Location

*Mark only one oval.*

Africa

Antarctica

Asia

Australia

Europe

North America

1. How many students attend the course?

*Tick all that apply.*

1-14

15-29

30-44

45-59

60-74

75-89

90-104

More than 104

Other:

1. What teaching methods are used in endodontic course? (it is possible to mark more than one answer) *

*Tick all that apply.*

Lectures

Seminars (mostly interdisciplinary discussion of content in smaller groups than in the lectures)

Tutorials= (are led by students with appropriate qualifications, supervised by members of the teaching staff and represent a possible form of regulated self-study by students)

Problem based learning

Independent study

Projects/ assignments

E-learning

Laboratory and practical learning

Videos

Manuals

Reading lists

Other:

1. In which year/years are the specific topics of the Endodontics program taught? (it is possible to mark more than one answer) *


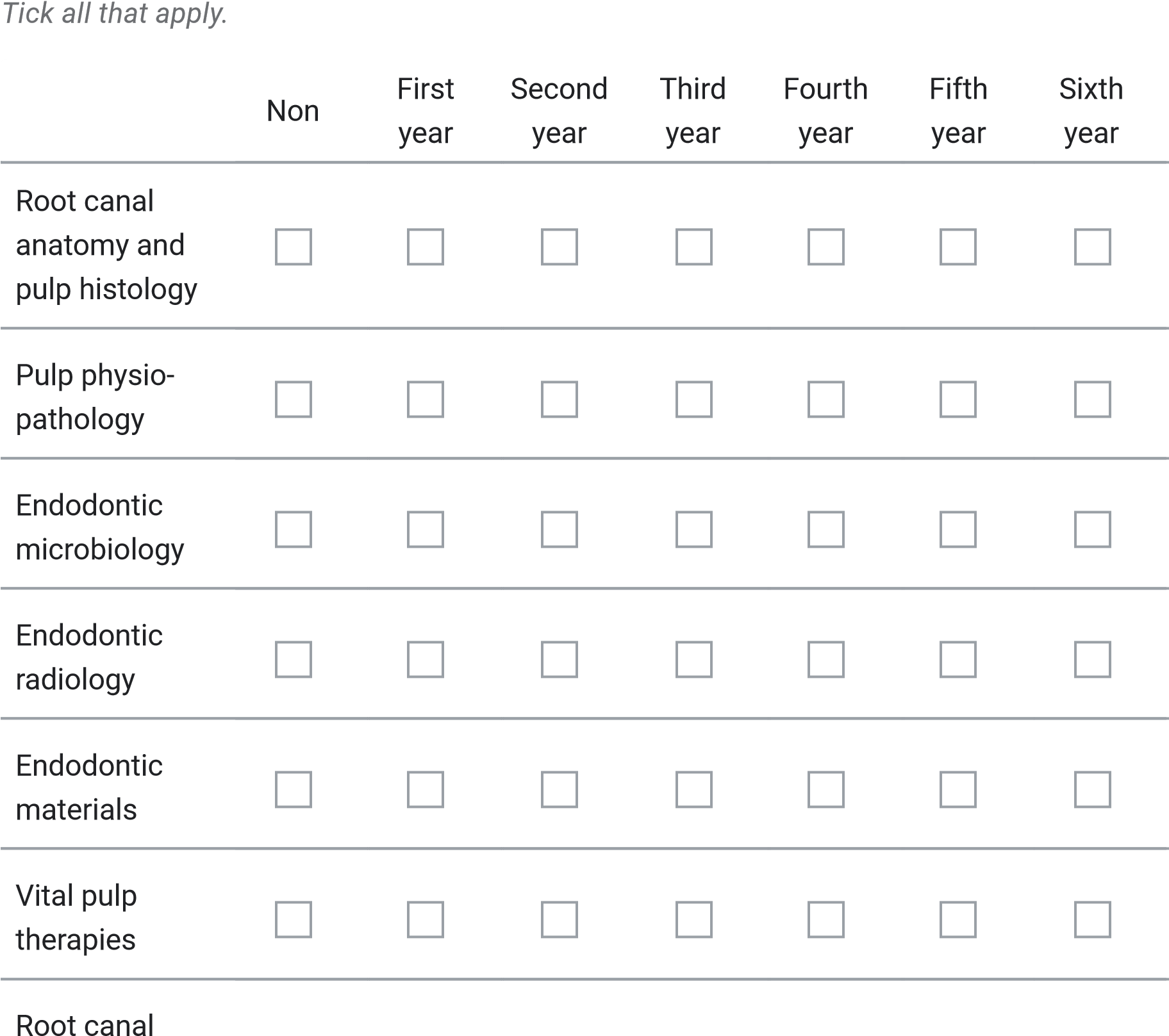


Treatment of immature teeth

Non-surgical endodontic treatment

Root canal retreatment

Surgical endodontic treatment

Endodontic regeneration

Restoration of endodontically teeth

Bleaching of endodontically treated teeth

Dental trauma

Endodontic emergencies

Perio-endodontic problems

Ortho-endodontic problems

**Preclinical training**

**All questions in this section are related to preclinical endodontic training**

1. In what year/years is preclinical endodontics taught? (it is possible to mark more than one answer) *

*Tick all that apply.*

First year

Second year

Third year

Fourth year

Fifth year

Sixth year

1. Is preclinical course taught as a separate course or in integration with other courses?

*Mark only one oval.*

it is a separate course

It is integrated with other courses

Other:

1. Which dental simulators is available for the students to perform preclinical training? (it is possible to mark more than one answer) *

*Tick all that apply.*

Traditional phantom head (manikin)

Computer-supported and Virtual reality dental simulators

No dental simulator

Other:

1. What are the total hours (1hour= 45 minutes) allocated to the ***theoretical*** part of preclinical endodontic training?

*Mark only one oval.*

1 hour

30 hours

45 hours

60 hours

75 hours

90 hours

Other:

1. What is the staff:students ratio during preclinical training?

*Mark only one oval.*

1:5

Up to 1:8

Up to 1:10 More than 1:10

Other:

1. What is the qualification of the majority of course contributors in preclinical training? (it is possible to mark more than one answer) *

*Tick all that apply.*

General dentistry

General dentistry with particular interest in endodontics

Restorative Dentistry Exclusively Endodontics

Other:

1. What types of root canals are used in preclinical training? (it is possible to mark more than one answer) *

*Tick all that apply.*

Canals in natural extracted teeth

Canals in commercial plastic teeth

Canals in 3D printed teeth

Canals in acrylic blocks with simple curves

Canals in acrylic blocks with S-shaped curves

Other:

1. What is the minimum number of canals that students perform root canal treatment during preclinical training?
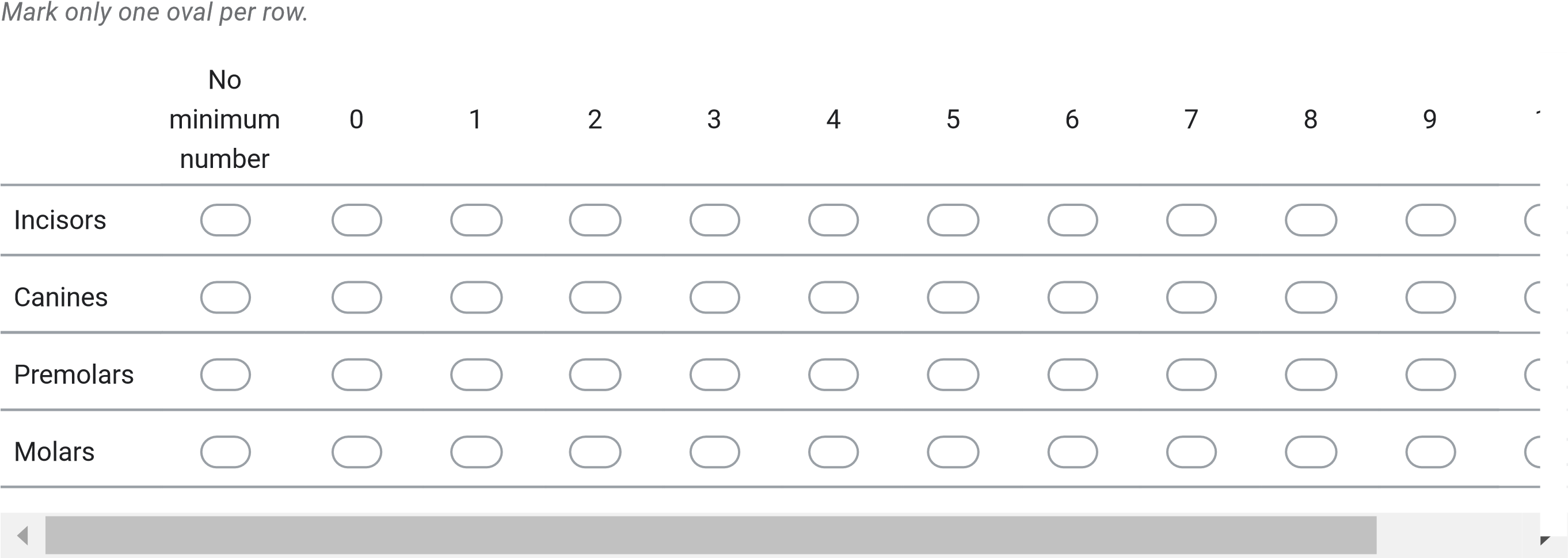

2. What is the minimum number of canals that students perform root canal ***retreatment*** during preclinical training?


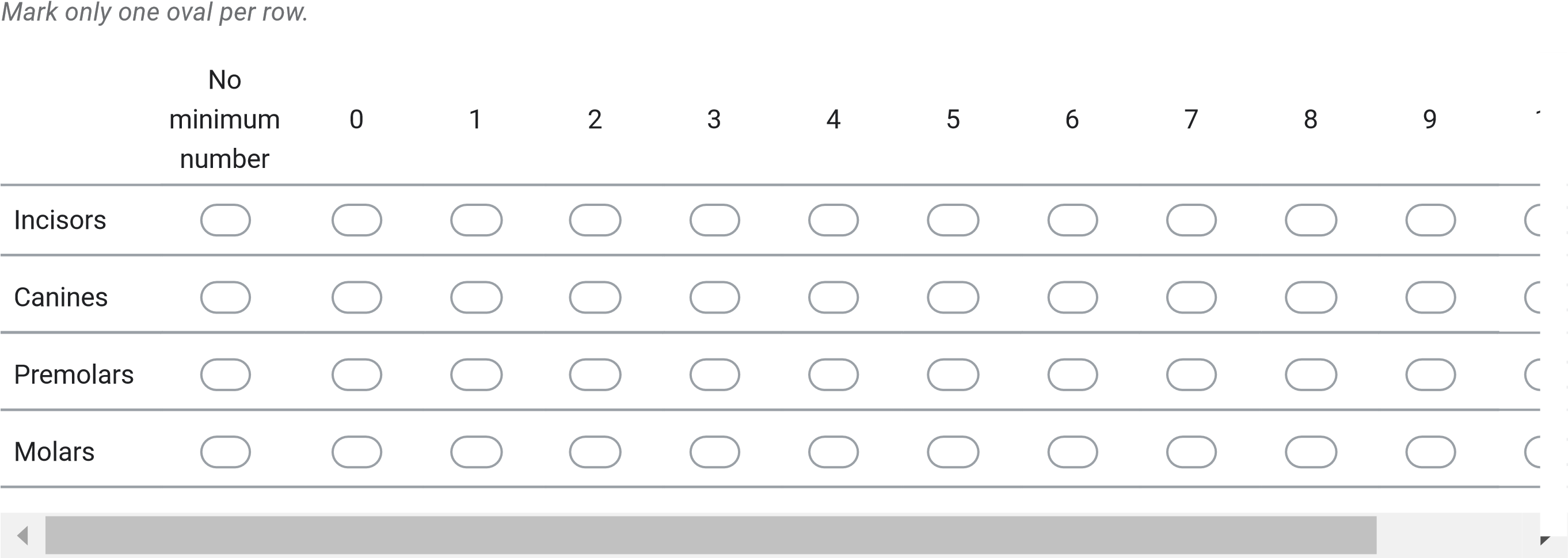


1. Which magnifying systems are used by students during pre-clinical training? (it is possible to mark more than one answer) *

*Tick all that apply.*

Magnifying systems are not used

Dental loupes

Microscope

Other:

1. Do students use ultrasonic instruments in preclinical training?

*Mark only one oval.*

Yes

No

Other:

1. What method of working length determination is used during preclinical training

*Mark only one oval.*

Radiographs only

Electronic apex locator only

Both radiographs and electronic apex locator

Other:

1. Which instruments are used by the students for root canal shaping during preclinical training? (it is possible to mark more than one answer) *

*Tick all that apply.*

Manual stainless instruments

Manual Nickel- titanium instruments

Engine driven rotary NiTi files

Engine driven reciprocating NiTi files

1. What is the main manual (hand) instrumentation technique of root canal preparation used by the students in preclinical training? (it is possible to mark more than one answer) *

*Tick all that apply.*

No manual technique used

Step back technique

Crown down technique

Balanced force technique

Standardized technique

Hybrid technique (step back and crown down techniques)

Other:

1. Which materials/ instruments are used by the students for removing Root Canal Filling Material in ***Retreatment*** during preclinical training? (it is possible to mark more than one answer) *

*Tick all that apply.*

Students do not perform retreatment

Gates Glidden drills

Peeso Reamers

Nickel-titanium rotary files

Hedstrom file (H-F)

Xylene

Chloroform

Eucalyptol

Halothane

Endosolv (Ethyl acetate)

None

Other:

1. Which irrigants are used by the students during preclinical training? (it is possible to mark more than one answer) *

*Tick all that apply.*

Sodium hypochlorite

Chlorhexidine

Ethylenediamide tetraacetic acid (EDTA)

Citric acid

Local anesthetic solution

Normal saline

Water

None

Other:

1. What is the main irrigation technique used by the students when performing root canal treatment in the preclinical training? (it is possible to mark more than one answer) *

*Tick all that apply.*

Passive needle irrigation

Manual agitation using gutta percha

Dynamic irrigation using sonic ultrasound energy

Dynamic irrigation using ultrasonic energy

Other:

1. What is the main method of root canal filling used by the students during preclinical training? (it is possible to mark more than one answer) *

*Tick all that apply.*

Single cone technique

Cold lateral compaction

Warm vertical compaction (Schilder technique)

Warm vertical compaction using continuous technique (Buchanan)

Thermoplastic injection technique

Carrier-based technique

Paste fillers

Thermo-mechanic compaction

Other:

1. Do students use advanced endodontic materials/technologies during preclinical training? (it is possible to mark more than one answer) *

*Tick all that apply.*

Mineral trioxide aggregate (MTA)

Biodentine

Bioceramic sealers

Cone beam computed tomography (CBCT)

Thermally treated nickel-titanium files Guided endodontic access technology

Other:

**Clinical training**

**All questions in this section are relate to clinical training**

1. What is the staff:students ratio during clinical training?

*Mark only one oval.*

1:5

Up to 1:8

Up to 1:10 More than 1:10

Other:

1. In what year/years is clinical endodontics taught? (it is possible to mark more than one answer) *

*Tick all that apply.*

First year

Second year

Third year

Fourth year

Fifth year

Sixth year

1. Is there a clinical area specifically assigned to Endodontics?

*Mark only one oval.*

Yes

No

Other:

1. What is the qualification of the majority of course contributors in clinical training? (it is possible to mark more than one answer) *

*Tick all that apply.*

General dentistry

General dentistry with particular interest in endodontics

Restorative Dentistry Exclusively Endodontics

Other:

1. What types of endodontic treatments do students perform during clinical training? (it is possible to mark more than one answer) *

*Tick all that apply.*

Non-surgical root canal treatment

Vital pulp therapy

Surgical root canal treatment

Regenerative endodontics

Bleaching of endodontically treated teeth

Retreatments

Students do not execute any treatment

1. What is the minimum number of canals that students perform root canal treatment during clinical training?


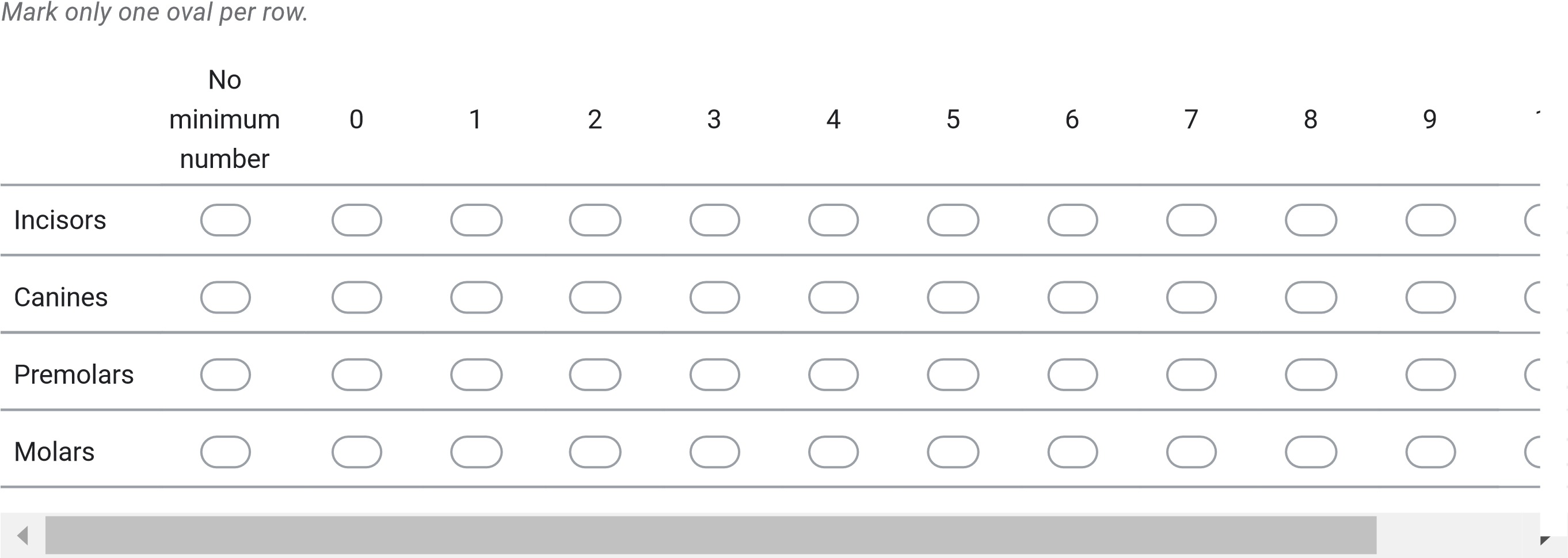


1. What is the minimum number of canals that students perform root canal ***retreatment*** during clinical training?


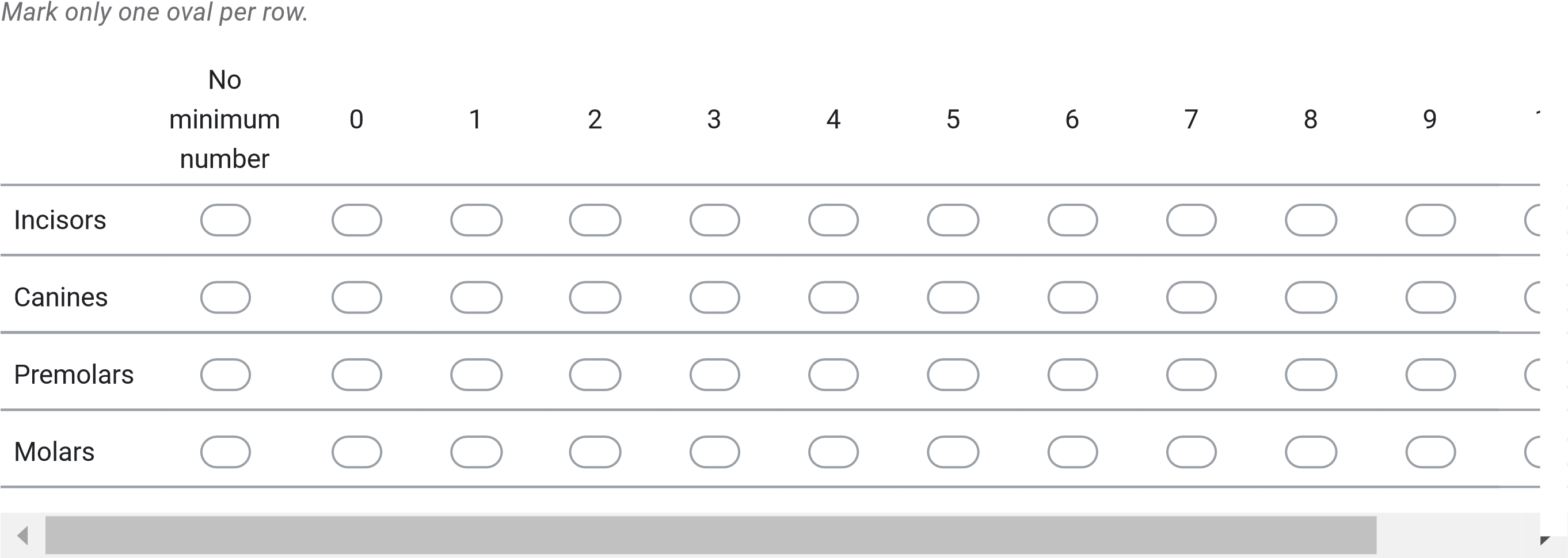


1. Which magnifying systems are used by students during clinical training? (it is possible to mark more than one answer) *

*Tick all that apply.*

Magnifying systems are not used

Dental loupes

Microscope

Other:

1. Do students use ultrasonic instruments in clinical training?

*Mark only one oval.*

Yes

No

Other:

1. What method of working length determination is used during clinical training?

*Mark only one oval.*

Radiographs only

Electronic apex locator only

Both radiographs and electronic apex locator

Other:

1. Which instruments are used for root canal shaping during clinical training? (it is possible to mark more than one answer) *

*Tick all that apply.*

Manual (hand) stainless instruments

Manual (hand) Nickel- titanium instruments

Engine driven rotary NiTi files

Engine driven reciprocating NiTi files

1. What is the main manual (hand) instrumentation technique of root canal preparation used by the students during clinical training? (it is possible to mark more than one answer) *

*Tick all that apply.*

No manual technique used

Step back technique

Crown down technique

Balanced force technique

Standardized technique

Hybrid technique (step back and crown down techniques)

Other:

1. Which materials/ instruments are used by the students for removing Root Canal Filling Material in ***Retreatment*** during clinical training? (it is possible to mark more than one answer) *

*Tick all that apply.*

Students do not perform retreatment

Gates Glidden drills

Peeso Reamers

Nickel-titanium rotary files

Hedstrom file (H-F))

Xylene

Chloroform

Eucalyptol

Halothane

Endosolv (Ethyl acetate)

None

Other:

1. Which irrigants are used by the students during clinical training? (it is possible to mark more than one answer) *

*Tick all that apply.*

Sodium hypochlorite

Chlorhexidine

Ethylenediamide tetraacetic acid (EDTA)

Citric acid

Ethanol

Local anesthetic solution

Normal saline

Water

None

Other:

1. What is the main irrigation technique used by the students when performing root canal treatment in the clinical training? (it is possible to mark more than one answer) *

*Tick all that apply.*

Passive needle irrigation

Manual agitation using gutta percha

Dynamic irrigation using sonic ultrasound energy

Dynamic irrigation using ultrasonic energy

Other:

1. What is the main method of root canal filling used by the students during clinical training? (it is possible to mark more than one answer) *

*Tick all that apply.*

Single cone technique

Cold lateral compaction

Warm vertical compaction (Schilder technique)

Warm vertical compaction using continuous technique (Buchanan)

Thermoplastic injection technique

Carrier-based technique

Paste fillers

Thermo-mechanic compaction

Other:

1. Which type of endodontic dressings (intracanal medicament) are used by the students during clinical training? (it is possible to mark more than one answer) *

*Tick all that apply.*

Calcium hydroxide

Ledermix

None – single visit treatment whenever possible based on different diagnosis

No medicament used

Other:

1. What is the type of restoration placed by the students after completion of root canal treatment in the clinical?

*Mark only one oval.*

Provisional (temporary) restoration (cement filling)

Definitive restoration (adhesive filling)

Other:

1. Do students use advanced endodontic materials/technologies during clinical training? (it is possible to mark more than one answer) *

*Tick all that apply.*

Mineral trioxide aggregate (MTA)

Biodentine

Bioceramic sealers

Cone beam computed tomography (CBCT)

Thermally treated nickel-titanium files Guided endodontic access technology

Other:

1. Do you follow a difficulty assessment form before assigning clinical cases to students?

*Mark only one oval.*

Yes, there is a difficulty assessment form (the American Association of Endodontics (AAE) difficulty assessment Yes, there is a difficulty assessment form (not AAE form).

No special difficulty assessment form).

No

Other:

1. What is the degree of case complexity (according to the American Association of Endodontics (AAE) difficulty assessment form) that allowed for the students to treat in the clinic?

*Tick all that apply.*

Uncomplicated cases with low difficulty.

Cases with moderate difficulty

Cases with high difficulty

**Assessment methods**

1. What is the assessment method used to assess the theoretical part of Endodontology? (it is possible to mark more than one answer) *

*Tick all that apply.*

Multiple choice (MCQs)

Written exam with free text

Oral examination

Objective structured Oral examination OSOE

Other:

1. What is the assessment method used to assess the practical part of Endodontology? (it is possible to mark more than one answer) *

*Tick all that apply.*

On patients

OSCE = Objective Structured Clinical Examination

In an alternative format (e.g., Mini-CEX = Mini-Clinical Evaluation Exercise, DOPS = Direct Observation of Procedural Skills)

On human extracted teeth

On industrially produced standardized simulation teeth made of plastic

Portfolio

Case presentation of clinical work

Other:
